# The algal selenoproteomes

**DOI:** 10.1186/s12864-020-07101-z

**Published:** 2020-10-07

**Authors:** Liang Jiang, Yiqian Lu, Lin Zheng, Gaopeng Li, Lianchang Chen, Maona Zhang, Jiazuan Ni, Qiong Liu, Yan Zhang

**Affiliations:** 1 College of Life Sciences and Oceanography, Shenzhen University, Shenzhen, Guangdong Province 518060 P.R. China; 2 Brain Disease and Big Data Research Institute, College of Life Sciences and Oceanography, Shenzhen University, Shenzhen, Guangdong 518055 P. R. China; 3 Shenzhen Bay Laboratory, Shenzhen, 518055 P. R. China

**Keywords:** Selenium, Selenoprotein, Algae, Evolution, Genomics

## Abstract

**Background:**

Selenium is an essential trace element, and selenocysteine (Sec, U) is its predominant form in vivo. Proteins that contain Sec are selenoproteins, whose special structural features include not only the TGA codon encoding Sec but also the SECIS element in mRNA and the conservation of the Sec-flanking region. These unique features have led to the development of a series of bioinformatics methods to predict and research selenoprotein genes. There have been some studies and reports on the evolution and distribution of selenoprotein genes in prokaryotes and multicellular eukaryotes, but the systematic analysis of single-cell eukaryotes, especially algae, has been very limited.

**Results:**

In this study, we predicted selenoprotein genes in 137 species of algae by using a program we previously developed. More than 1000 selenoprotein genes were obtained. A database website was built to record these algae selenoprotein genes (www.selenoprotein.com). These genes belong to 42 selenoprotein families, including three novel selenoprotein gene families.

**Conclusions:**

This study reveals the primordial state of the eukaryotic selenoproteome. It is an important clue to explore the significance of selenium for primordial eukaryotes and to determine the complete evolutionary spectrum of selenoproteins in all life forms.

## Background

Selenium (Se) is an essential trace element for many organisms, from bacteria to humans. This micronutrient plays essential roles in redox homeostasis involved in various cellular processes and may provide numerous health benefits, such as preventing cancer and heart disease, boosting immune function, and regulating the aging process [1–5]. The main biological form of Se is selenocysteine, the 21st amino acid in the genetic code, which is encoded by the UGA codon and then cotranslationally incorporated into selenoproteins. In eukaryotes, the mechanism of Sec insertion in response to UGA involves (i) a cis-acting Sec insertion sequence (SECIS) element, which is a highly specific stem-loop structure located in the 3′-UTR of selenoprotein mRNAs, and (ii) several trans-acting factors dedicated to Sec incorporation, including the eukaryotic Sec synthase (SecS), eukaryotic Sec-specific elongation factor (eEFSec), selenophosphate synthetase 2 (SEPHS2), O-phosphoseryl-tRNA^[Ser]Sec^ kinase (PSTK), SECIS binding protein 2 (SBP2) and additional proteins [6–10].

Identification of full sets of selenoproteins in organisms (called selenoproteomes) is key for understanding the biological roles of Se. In recent years, based on the critical features detected in SECIS elements and the conservation between selenoproteins and their cysteine (Cys)-containing homologs, several bioinformatics algorithms have been successfully developed for the prediction of selenoprotein genes in eukaryotic genomes [11–15]. With these approaches, a number of selenoproteins have been predicted and further experimentally identified in a variety of eukaryotes, such as animals and several unicellular organisms [16–18]. For example, 25 and 24 selenoproteins have been reported in humans and mice, respectively. Larger selenoproteomes in vertebrates were found in aquatic organisms, such as zebrafish, which contain 38 selenoprotein genes [16]. Previously, we analyzed the selenoproteomes in certain metazoans by using our SelGenAmic algorithm [19, 20]. These studies have provided important clues for understanding the occurrence and evolution of selenoproteins as well as their relationship with ecological environments, especially during metazoan history [21–23]. However, it is unclear whether similar or different evolutionary trends occurred in other eukaryotic lineages.

Compared with multicellular animals and higher plants that appeared approximately 400 or 500 million years ago, eukaryotic algae were reported to have originated more than 1.5 billion years ago and constitute a very diverse group of organisms inhabiting a vast range of ecosystems [24, 25]. Analysis of the distribution and evolution of eukaryotic algal selenoproteins may not only delineate the primitive state of Se utilization in eukaryotes but also elucidate the complex evolutionary history of Se utilization in such a collection of extremely diverse organisms. Previous studies have identified selenoprotein genes in a very limited number of algae, such as *Aureococcus anophagefferens* and *Chlamydomonas reinhardtii* [26, 27]. Very recently, Liang et al. examined the Sec machinery and selenoproteins in 33 algal species belonging to several groups of Archaeplastida, which provides preliminary clues for their evolutionary dynamics in this lineage of photosynthetic eukaryotes [28]. Considering that the algal world contains a variety of groups that do not belong to Archaeplastida, such as Alveolates and Rhizaria, and that genomic or transcriptomic data are available for many algal species in which Se utilization has not been explored, a comprehensive study on the distribution and evolution of selenoproteins is urgently needed at a much larger scale. It is also interesting to investigate the relationship between environmental factors and Se utilization in algae.

This study reported a comprehensive survey of the algal selenoproteomes in more than 100 algal species based on genomic and/or transcriptomic data. The composition, evolution, and properties of algal selenoproteomes were systematically analyzed using different approaches. Potential interactions between environments and selenoprotein families were also investigated. Overall, these data provide novel insights into selenoprotein function and evolution in a widespread, abundant, and ecologically important group of unicellular organisms.

## Results

### Composition and distribution of the algal selenoproteome

**Fig. 1:**
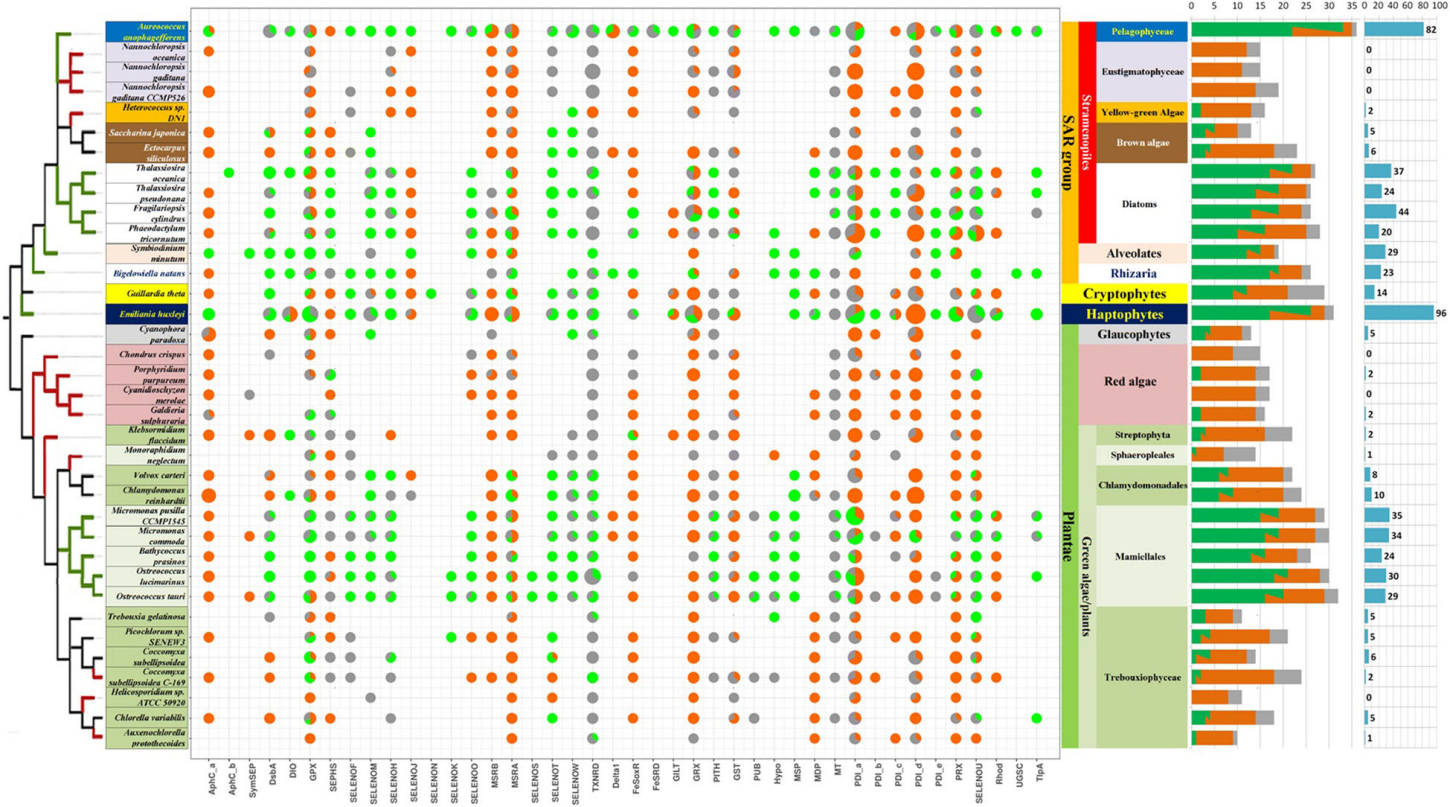
Distribution of algal selenoproteomes. Selenoprotein families predicted based on the genomic sequences of 36 algal species. The taxonomic tree of these organisms is shown on the left (based on ref [29, 30]). In the tree, a green branch indicates a high-level selenium-containing organism (with a number of selenoproteins in a single species > = 20), and a red branch represents a low-level organism (with a number of selenoproteins in a single species <= 2). On the right, the taxonomic classification of different groups of algae is shown in different colors. The presence or absence of a selenoprotein and/or its homologs in each organism is highlighted in the pie graphs: The green, orange, and gray colors represent selenoproteins, Cys-containing homologs, and homologs containing other residues, respectively. The sizes of the whole pie and each sector represent the number of genes in the corresponding groups. The first bar on the right shows the number of selenoprotein families in different algae. The meaning of the various colors in the cylinder is consistent with the color in the pie chart matrix (green: Sec, orange: Cys, gray: others). While the length of the column represents the total number of protein families in each species, the two-color column (green and orange) indicates that the multiple protein families in the species include both Sec-containing and Cys-containing members. The rightmost blue bar chart shows the number of selenoprotein genes found in the genome of each species

We predicted more than 1000 selenoprotein genes from genomic (36 organisms) and/or transcriptomic (including EST) datasets of 137 algal species (detailed information about these organisms is shown in Table S1 in Supplementary file 1). The distribution of selenoproteins and their Cys-containing homologs in these organisms is shown in Figure S1 in Supplementary file 1. Details about these selenoprotein genes are available at the SPDB database website (http://www.selenoprotein.com). Algal selenoproteins can be identified in textual information by searching for the species or selenoprotein family name or can be identified by their sequence using a web blast tool [33]. For each selenoprotein gene, information such as the nucleic acid sequence, amino acid sequence, SECIS element, gene splicing structure, and EST alignment information was recorded. A detailed description of this database is shown in Figures S10, S11, S12, S13, S14 and S15. Considering that the majority of organisms examined here had only transcriptomic data, the possibility that additional selenoprotein genes were not sequenced in some of these organisms could not be neglected. Figure 1 shows the distribution of different selenoproteins and their homologs in the 36 algae with genomic sequences.

According to the taxonomic classification of algae [29–32, 34, 35], we divided these species into Plantae (including Green algae, Red algae, and Glaucophytes), the SAR group (including Stramenopiles, Alveolates, and Rhizaria), Cryptophytes and Haptophytes. The majority of these algae (34 out of 36) belong to Plantae and the SAR group. The composition of the algal selenoproteome varied dramatically among different taxonomic groups, including a group of six species in which no selenoprotein gene could be detected (Fig. 1 and Figures S1 and S3). However, in certain lineages, the number of selenoproteins appeared to be more stable. For example, all the algae species possessing larger selenoproteomes (containing more than 20 selenoproteins, as shown by the green branches in Fig. 1) were found in Mamiellales and Diatoms, whereas the algae having smaller selenoproteomes (less than 2 selenoproteins, as shown by the red branches in Fig. 1) were detected in red algae and Eustigmatophyceae.

In Plantae, the size of the selenoproteome varied significantly among different organisms. Red algae and glaucophytes had very small selenoproteomes, including two organisms (*Chondrus crispus* and *Cyanidioschyzon merolae*) in which no selenoprotein genes could be detected. Among green algae, Mamiellales species had the largest selenoproteomes (> 20 selenoproteins), whereas Sphaeropleales, Streptophyta, and Trebouxiophyceae had the smallest selenoproteomes (0–5 selenoproteins). Compared with other algae, Streptophyta is evolutionarily closer to land plants. Although the only organism with sequenced genomic data found in this clade, *Klebsormidium flaccidum*, contains only two selenoprotein genes, more selenoprotein genes were detected in some other streptophytes using EST data, such as *Nitella hyalina* and *Chaetosphaeridium globosum*, which are thought to be closer to higher-level plants than *K. flaccidum* (Figure S1).

The distribution of known selenoproteins in the SAR group was also highly variable. Stramenopiles are the largest group of SAR and include Diatoms, brown algae, yellow-green algae, Phaeophyceae, and Eustigmatophyceae. In *A. anophagefferens*, a pelagophyte, 82 selenoprotein genes belonging to 33 families were found. It has been previously reported to have the largest eukaryotic selenoproteome [26]. The number of selenoprotein genes in diatoms varied from 20 to 44, which is similar to the size of the selenoproteomes in the Mamiellales order of green algae. Brown algae and yellow-green algae had much smaller selenoproteomes (5–6 selenoprotein genes). Moreover, no selenoprotein gene was detected in Eustigmatophyceae. Alveolates and Rhizaria are the other two groups of the SAR group; we detected 29 and 23 selenoprotein genes in *Symbiodinium minutum* (Alveolates) and *Bigelowiella natans* (Rhizaria), respectively.

Two additional algae species with sequenced genomes are *Guillardia theta* (Cryptophyte) and *Emiliania huxleyi* (Haptophyte). Fourteen selenoproteins belonging to 12 families were detected in *G. theta*. Surprisingly, a total of 96 selenoprotein genes were identified in *E. huxleyi*, which is the largest selenoproteome within all organisms discovered so far; the selenoproteins belong to 25 different families. Such a large number of selenoprotein genes might be related to the high repetition rate of the genome of *E. huxleyi* [36].

Forty-two selenoprotein families were predicted in algae. Many algal selenoproteins have homologous proteins containing no Sec residues, and the most common substitution involves the replacement of Sec by Cys (hereinafter referred to as Cys-homologous). In addition, there are many other homologs of selenoproteins in which the corresponding position of Sec contains neither Sec nor Cys (hereinafter referred to as Other-homologs). The homologous proteins (Cys-homologs and Other-homologs), although they are probably not related to Se metabolism, may function similarly due to their sequence similarity. More importantly, they contain information on the evolution of selenoprotein families. Therefore, we also included Cys-homolog and Other-homolog data when analyzing the evolution and distribution of the selenoprotein family.

Figure S2 shows the distribution of algae containing different selenoproteins and/or their homologs. Considering the distribution of all types of homologous proteins (Sec-containing, Cys-homologs, and Other-homologs), the PDI_a and TXNRD families are present in all 36 algal genomes, and GPX and GRX are also present in 35 species (Figure S2A). Therefore, these protein families may be essential for the majority of algae. However, the proportion of Sec-containing proteins is different, as PDI_a and GRX are present in the Cys-containing form in most algae, while GPX and TXNRD are mainly present in the Sec-containing form. Figure S2B shows the ranked distribution of selenoproteins (Sec-containing) in different algae. Sec-containing forms of four selenoproteins, GPX, SELENOU, SELENOT and TXNRD, could be found in more than half of the 36 genomes and are the most widely distributed selenoproteins in algae.

Figure S2C shows the proportion of Sec-containing members in each protein family. Some selenoprotein families, such as MSP, SELENOK, SELENOS, USGC, AhpC_b, SELENON, and FesRD, are found almost exclusively in the form of Sec-containing proteins. In addition, 80% of DIO, TlpA, SELENOW, and Hypo family members are Sec-containing proteins. These selenoproteins have fewer non-Sec-containing homologous proteins, indicating that their function is more dependent on Se metabolism in algae. In contrast, members of some other selenoprotein families, such as MsrB, PDI_d, AhpC_a, and GST, are found as Cys-homologous or Other-homologous proteins in nearly 90% of algae genomes.

### Identification of novel selenoproteins

**Fig. 2:**
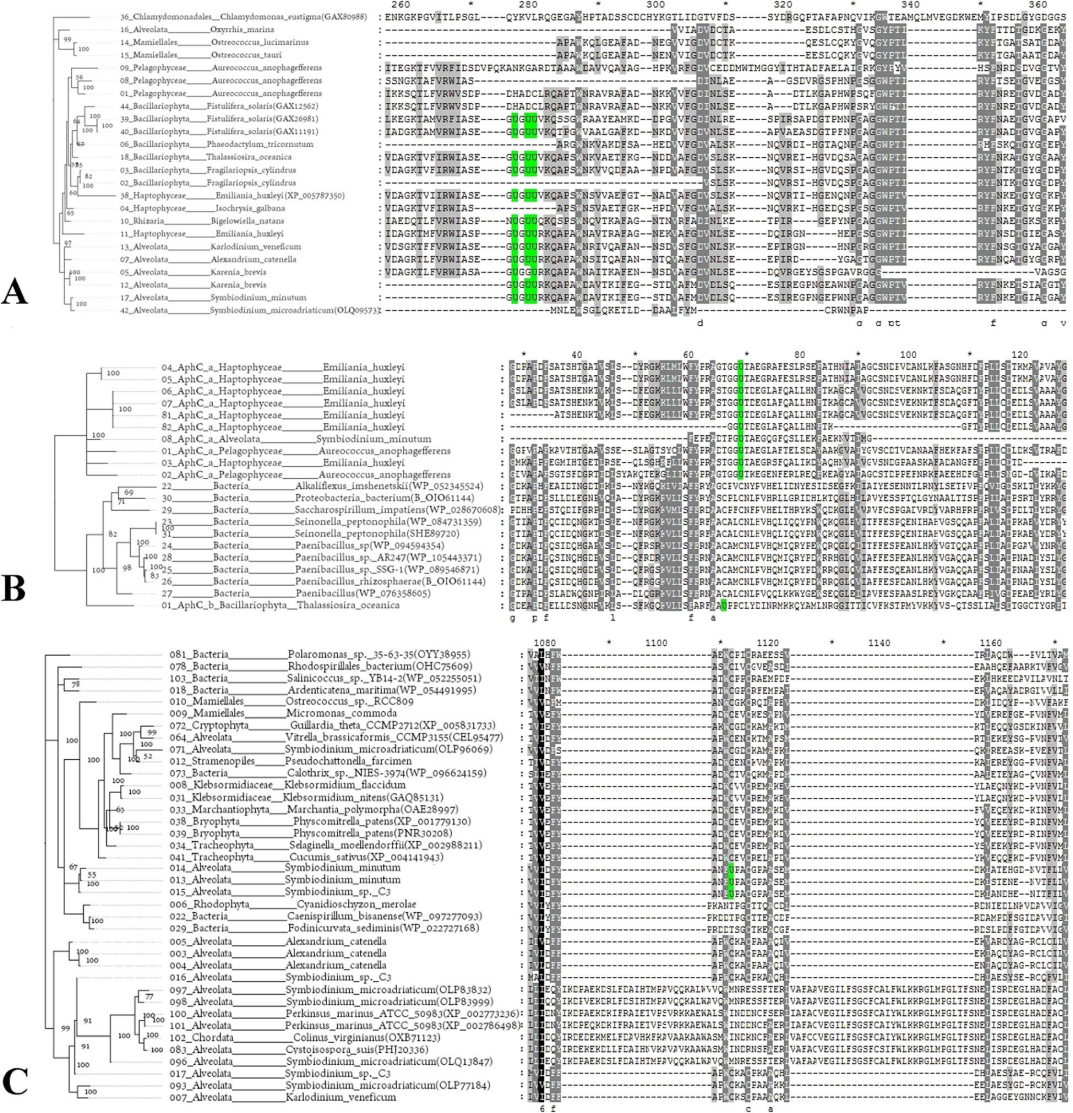
Multiple sequence alignment and phylogenetic analysis of novel selenoproteins. **a** PDI_e, **b** AhpC, **c** SymSEP. The Sec residue is marked with a green background. The sequence numbers, phyla names, and organism names are shown on the left, and the sequences from the NR database are shown with their accession IDs in brackets

In this study, three novel selenoprotein families were found in different algae (Figs. 1 and 2).

#### PDI_e

We found a large number of PDI-like protein genes in algae. The thioredoxin-like fold domain can be detected in most of these proteins. Therefore, their functions may be related to redox regulation. Based on the amino acid sequences surrounding the Sec residue, PDI sequences could be divided into five subfamilies (Figure S3): PDI_a, PDI_b, PDI_c, and PDI_d, which contain only one Sec, and PDI_e (as named in this study), which was found to have three neighboring Sec residues that formed a GUGUU motif (Fig. 2a). This is the first study to discover a selenoprotein with two consecutive Sec residues. Because of this Sec-Sec sequence, we considered PDI_e as a novel selenoprotein (the EST sequence alignment and predicted SECIS elements of PDI_e in several organisms are shown in Figure S4). We speculate that the selenoprotein synthesis system of organisms containing PDI_e is sufficient to meet the requirements of decoding continuous TGA codons. Correspondingly, the number of selenoproteins in several PDI_e-containing algae was also abundant (Fig. 1, Figures S1 and S3). Even in some PDI_e-containing algae without the relevant genomic sequences, many selenoproteins could also be detected. For example, in *Isochrysis galbana,* 17 selenoproteins from 14 families were found in 6432 assembled Est contigs, and in *Karenia brevis,* 29 selenoproteins from 17 families were found in 29,618 assembled Est contigs.

We found a total of 12 PDI_e genes in 10 different algae. They are mainly distributed in Haptophyceae and the SAR group. The loss of the GUGUU motif occurred in the homologous proteins of *Fistulifera solaris*. There was no Sec-containing PDI_e sequence in the Plantae group, and only non-Sec-containing sequences homologous to PDI_e were detected. In Fig. 2a, in addition to the PDI_e proteins found in algae, the proteins found in the NR database that have sequence similarity to PDI_e are also shown. The results show that there is no protein homologous to PDI_e in bacteria, fungi, or other multicellular eukaryotes, so we conclude that this is a selenoprotein found only in single-celled eukaryotic algae.

#### AhpC_b

Two families of selenoproteins containing AhpC_TSA domains could be found in algae, AhpC_a and AhpC_b. AhpC_a was detected in almost all algae species, but most of the corresponding proteins were Cys-containing homologs. The Sec-containing AhpC_a was present in only three algal species: *A. anophagefferens*, *E. huxleyi,* and *S. minutum*. AhpC_b was found in *Thalassiosira oceanica*. There is a detectable similarity between AhpC_b and AhpC_a, but the Sec-flanking sequences are significantly different. In the NR database, we found several proteins homologous to AhpC_b. However, interestingly, all of these homologs were found in prokaryotic organisms and in Cys form. Figure 2b shows the phylogenetic tree and multiple alignment of amino acid sequences of AhpC_b, their closest homologs from prokaryotic species, and all Sec-containing AhpC_a in algae. As shown in Fig. 2b, the UxxC (CxxC) motif of AhpC_b and other prokaryotic homologs is different from the TGGUT motif of AhpC_a. Because of the difference between the key motif and the whole sequences, we considered AhpC_b as a novel selenoprotein (the SECIS element is shown in Figure S4). We speculate that it potentially originated from a prokaryotic ancestor by horizontal gene transfer because no similar eukaryotic sequence was found. Due to its AhpC_TSA domain, the function of AhpC_b may be related to antioxidation.

#### SymSEP

We found a selenoprotein family that was present in Symbiodinium phyla only in the Sec-containing form. We named it SymSEP. Four SymSEP selenoproteins were found among the genomic sequences and Est contigs from 2 species, *Symbiodinium minutum* and *Symbiodinium sp. C3*. The SECIS elements were detected and are shown in Figure S4 in supplementary file 1 (in the unpublished data, we also found a SymSEP sequence in *Symbiodinium microadriaticum*).

A phylogenetic tree and multiple sequence alignment of SymSEP-homologous proteins are shown in Fig. 2c. The figure shows all proteins similar to SymSEP found in all 137 algal sequences. Other similar proteins detected in the NR database are also included. As shown, the Sec-containing form of the protein is only present in the Symbiodinium phyla. Cys-containing homologs contain CxxC motifs that are widely distributed in a variety of eukaryotic algae and bacteria. In addition, there are two branches that do not contain either Sec or CxxC motifs. Based on the phylogenetic tree in the figure, we speculate that SymSEP first originated from prokaryotes in the form of a Cys-containing protein and only became a Sec-containing protein in Symbiodinium phyla after differentiation. The Trx-like domain was also detected in its coding region, suggesting that the function of SymSEP is related to redox regulation.

### Substitution of Sec

Sec is within the functional core site of the selenoprotein, and its codon is the termination codon TGA. Mutations in the codon result in the conversion of Sec into other amino acids, such as Cys (TGC, TGT) and Trp (TGG). Compared to that of Sec, their codon is only different at the third base. Among the various amino acids, the properties of Cys and Sec are the most similar, and most of the selenoproteins have homologous proteins in which Sec is substituted by Cys. The substitution of Sec by Cys is an important event in the evolution of selenoproteins.

As the correct translation of Sec-TGA requires complex synthetic systems, such as the SECIS structure located downstream of the coding region, the change from Cys to Sec is theoretically more difficult than that from Sec to Cys. The traces left by this transformation in the SECIS structure found downstream of the Cys-containing gene were previously reported. We also found a SECIS in a Cys-containing PRX from *S. minutum* (see attached Figure S5ABC in supplementary file 1). More interestingly, we found a pair of GRX genes in *Fragilariopsis cylindrus*. Their sequences are highly similar (positive > 80%), but one is Sec-containing, whereas the other is Cys-containing. Analysis of these two GRXs revealed a typical Sec-Cys substitution event. Most algae contain Cys-containing GRX, and Sec-containing GRX is only found in several selenoprotein-rich species from the SAR group and haptophytes. No Sec-containing GRX could be found in the Plantae group. Phylogenetic analysis of algae GRX revealed that the Sec-containing protein was clustered within a subtree which is partly shown in the Figure S6A. It can be inferred from the phylogenetic tree that most of the Sec-containing GRX have a common ancestor (except 001, 002, and 006). However, in the subtree branch, there are also a few Cys-containing homologous genes, which may undergo Sec-to-Cys changes. The Cys-containing GRX and Sec-containing GRX of *Fragilariopsis cylindrus* highlighted in Figure S6A have a common parental node; in other words, their differentiation has only recently occurred. More interestingly, the flanking genomic sequences of the two GRXs are homologous (see Figure S5D). Therefore, we hypothesize that these two GRXs may be derived from the same Sec-containing ancestral gene, in which genomic-level replication events occurred in this species or its related ancestors. The original single GRX gene was duplicated into two copies, and in one of the copies, Sec was converted into Cys due to a mutation. This is the first discovery of a genomic replication event associated with Sec-Cys substitution.

As we discussed above, the specific TGA decoding method and the complex synthesis system of selenoproteins make it very difficult for Cys to change into functional and genetically retainable Sec in terms of evolutionary history. However, in specific situations, the Cys-to-Sec mutation occurs in species with a functional selenoprotein synthesis system, and it occurs in a coding region upstream of a functional SECIS sequence; this change could be achieved. Then, the mutation will produce a decodable TGA-Sec codon. If the protein with the Cys-to-Sec change still has complete or partial function and allows the species to survive and breed, then it will be retained as a functional gene. Such events have been previously reported in several selenoproteins, especially those containing multiple Sec residues, such as SELENOP and several SELENOW proteins. In this study, we have found several new examples of Cys-to-Sec events. We previously found a SELENOW protein with 2 Sec in a UxxU motif in amphioxus, while in other SELENOW proteins, only one Sec was found in the CxxU motif. Interestingly, another UxxU-type SELENOW was found in this work (from *Ostreococcus lucimarinus*). The multiple sequence alignment of these SELENOW sequences is shown in Figure S7 in supplementary file 1. Another example of a Cys-to-Sec mutation was found in the SELENOJ family. SELENOJ was first discovered in vertebrates and was thought to exist only in multicellular animals [37]. Interestingly, multiple SELENOJ selenoproteins and Cys-containing homologs were detected in algae, including one sequence containing 2 Sec residues from *Alexandrium tamarense* (Figure S6B). In this 2-Sec-containing SELENOJ protein, the first Sec was also present in several algae and animals. The second Sec was only found in the EST sequences of *A. tamarense.* Therefore, it could be potential evidence of the Cys-to-Sec evolution event, which could lead to a novel selenium-related function due to the new position of Sec.

**Fig. 3:**
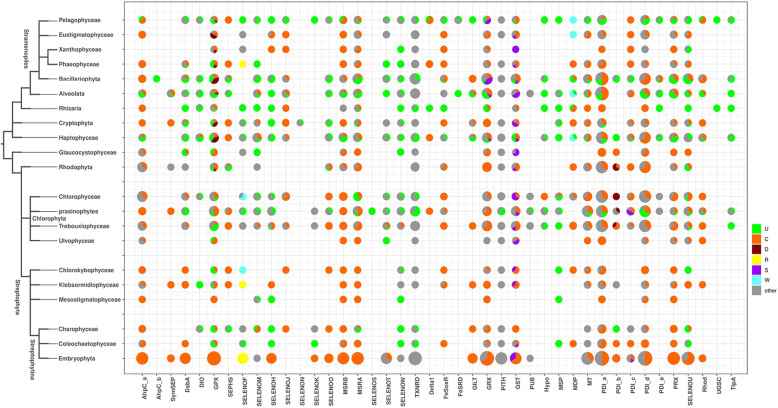
Substitution of Sec with other amino acids in algal selenoproteins. Statistics on the substitution of Sec in members of the selenoprotein family in all algae and related evolutionary phyla based on 137 algal sequences and the NR database. The size and proportion of the pie chart in the figure schematically show the number of genes of various types in each evolutionary phylum. Different colors represent the type of amino acid at the position containing Sec, and the meaning of the color is shown in the legend on the right

In addition to Cys homologs, we searched for non-Cys-containing homologs from 42 selenoprotein families in 137 algal datasets and the NR database. In these Other-homolog protein sequences, the local region corresponding to the position of the Sec motif was changed into other motifs. SELENOF is one of the earliest discovered animal selenoproteins [38]. It is mainly found in the Sec-containing form in multicellular animals and exists in the form of Cys homologs in only a few invertebrates (Arthropoda, Ecdysozoa, etc.) [39–41]. SELENOF is also widely distributed in algae, and the Sec-containing algal SELENOF protein contains the same CxU motif as the animal SELENOF protein. Interestingly, there is no Cys homolog of SELENOF found in algae. Instead, other homologs with other motifs were found in various algae. Their CxU motifs are converted into CMR in terrestrial plants and certain algae and into DQW in some green algae (Figure S6C). In addition, the Sec motif has undergone significant changes in some SELENOF proteins, resulting in the loss of local conservation, such as in SELENOF in *Micromonas commoda*. Despite the loss of the Sec-containing motif, these other homologs are still preserved and functional in the algal genomes of many different evolutionary domains, indicating that SELENOF has more functions not related to Se. Figure 3 shows the distribution of Sec-containing, Cys-homologous and Other-homologous proteins in the various evolutionary domains of eukaryotic algae (including terrestrial plants) in 42 selenoprotein-containing families of algae. In the GPX, GRX, GST, MDP, PDI, and other families, the core Sec motif has also become a non-Sec motif. In addition, the figure also shows the distribution of homologous selenoprotein proteins in terrestrial plants. Although there is no Sec-containing protein, most of the homologous proteins of unicellular algal selenoproteins are found in terrestrial plants. The phyla of terrestrial plants, such as Charophyceae (*Nitella hyalina*) and Coleochaetophyceae (*Chaetosphaeridium globosum)*, have a greater number of selenoproteins, suggesting that the loss of selenoproteins in terrestrial plants may have occurred in later geological ages.

### Selenoprotein gene clusters and fusion genes

**Fig. 4:**
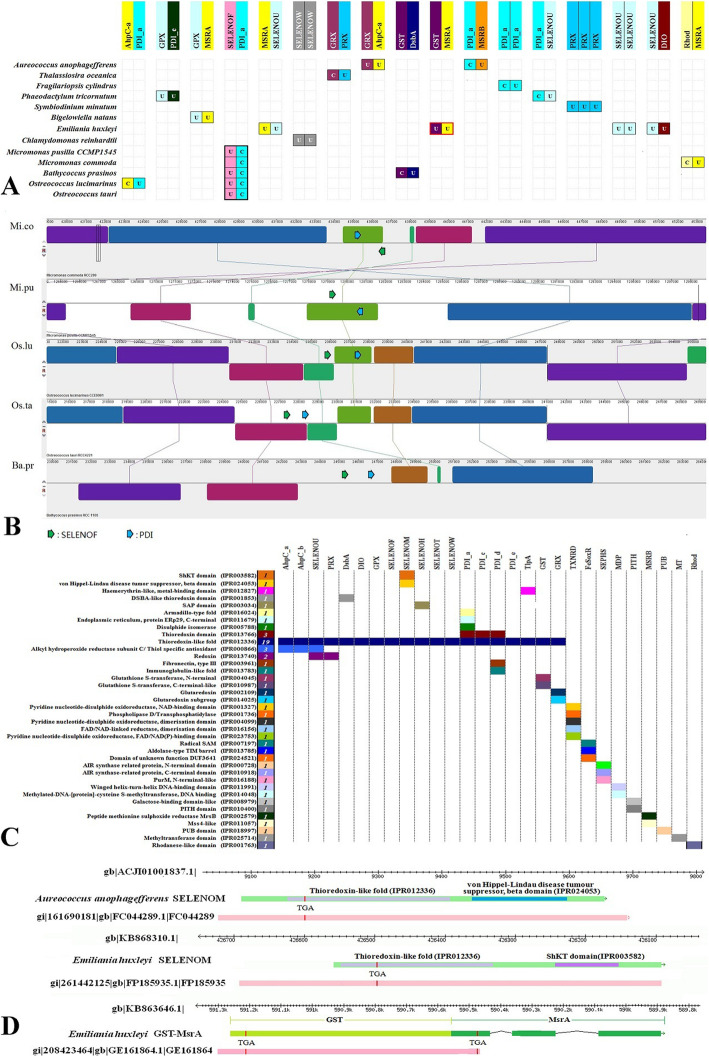
Gene clustering and fusion of algal selenoproteins. **a** Matrix of gene clusters of algal selenoproteins. A matrix cell composed of two or more colored boxes is a gene cluster. The colored box and the label on top indicate the family of the gene in the cluster. The U or C in the box represents the Sec or Cys form of the gene in the cluster. The species names are labeled on the left. **b** Genomic synteny of sequences containing the SELENOF-PDI_a gene cluster. **c** Conserved domain distribution matrix of algae selenoproteins. The abbreviation of each selenoprotein family is labeled at the top. The name and IPR id of the conserved domain are marked on the left. The number in the colored box next to the domain name indicates how many selenoprotein families contain the domain. The colored box in the matrix indicates that the corresponding domain has been detected in the selenoprotein family on the top. **d** Gene structure of fusion selenoprotein genes. The ruler on the top shows the genomic location. The arrow on the green box indicates the strand of the gene. The position of the EST matching the genome sequence is shown by the pink box

Genetic recombination, transposition, or whole-genome duplication can result in changes in the genomic location of the DNA fragment. These events may lead to clustering or fusion of genes. Previously, we reported clusters of selenoprotein genes in several invertebrate genomes, which might suggest a functional correlation between them [42–45]. Here, selenoprotein clusters were also observed in algae. Figure 4a shows the type and presentation of clusters in different algae. The gene structure and position of these clusters are shown in Figure S8 in supplementary file 1. As we can see from Fig. 4a, the clustering of selenoprotein genes was only found in 13 species. It is mostly found in *E. huxleyi*. The most frequently found selenoprotein families were MSRA and SELENOU. Among them, the SELENOF-PDI_a pair is the only species-cross cluster we detected, which suggests that the function of SELENOF is correlated with PDI in Mamiellales. Moreover, genome synteny is also detected in Mamiellales algae (shown in Fig. 4b) flanking these SELENOF-PDI pairs. Not all Mamiellales selenoprotein gene clusters have such a cross-species distribution, including AhpC_a-PDI_a, GST-DsbA, and Rhod-MSRA, which is only found in specific Mamiellales genomes. Considering genomic collinearity, we speculate that the genomic fragment in which SELENOF-PDI_a is located may have important functional or structural conservation in microalgae. Although the Sec motif was lost, the genomic level conservation in *Micromonas commoda* was retained. In addition, three clusters were composed of the same selenoprotein genes: two SELENOW genes in *Chlamydomonas reinhardtii*, two SELENOU genes in *Emiliania huxleyi,* and three PRX genes in *Symbiodinium minutum*. The adjacency of these gene locations in the genome indicates that they potentially originate from the duplication and differentiation of the same ancestor gene.

Recombination or transposition events, which occur within the coding region of a gene, may result in the truncation or fusion of genes. We scanned the conserved domains of all algal selenoproteins. Figure 4c shows that a total of 36 domains were detected in 29 algae selenoprotein families, and domain alignment diagrams for all selenoprotein families are provided in family page of Selenoprotein Database. The most frequently detected domain in algae selenoproteins was the Trx-like domain, which was present in approximately half (20) of the algal selenoprotein families. All of them are functionally related to the thiol/disulfide redox system, such as AhpC, PRX, PDI, DsbA, GPX, GRX, and GST. Other Trx-like-containing families, such as DIO, SELENOF, SELENOM, SELENOH, SELENOT, SELENOW, SELENOU, SELENOL and TlpA, also have oxidoreduction-related functions. In several selenoproteins, such as PITH, rhodanese, MSRA, and MSRB, no Trx-like domain could be detected; however, some of them have been reported to be functionally related to the oxide reduction process of sulfur. The PITH selenoprotein contains the proteasome-interacting thioredoxin domain. The rhodanese-like selenoprotein is likely to be a sulfur transferase involved in cyanide detoxification. MSRA and MSRB are widely present in animals and are related to the reduction of methionine sulfoxide [46, 47]. Another important function is also associated with algal selenoproteins. The hemerythrin metal-binding domain is found in the algae TlpA selenoprotein, which suggests its oxygen-binding function [48]. The iron-sulfur cluster binding-related catalytic activity could be indicated by the domains found in the FeS-oxidoreductase and reductase [49]. The methylated-DNA-[protein]-cysteine methyltransferase selenoprotein (MDP) is related to the biological process of DNA repair [50–52].

As shown in Fig. 4c and d, novel domain fusions were detected for several selenoprotein families in certain algae, including a SELENOM protein fused with the pVHL (Von Hippel-Lindau disease tumor suppressor beta domain) domain (*Aureococcus anophagefferens*), another SELENOM protein fused with the ShKT peptide toxin domain (*Emiliania huxleyi*), and a fusion protein of two selenoproteins (*E. huxleyi*). Their coding regions were found in both genomic and EST sequences. The multiple sequence alignment is shown in family page of Selenoprotein Database. As pVHL was previously reported as the substrate recognition component of an E3 ubiquitin ligase complex [53], it is possible that the SELENOM with the pVHL fusion potentially has a function related to tumor suppression [53]. Moreover, considering that the ShKT domain is often found in the anemone toxin protein, whose function is related to that of potent inhibitors of K(+) or iron channels, the fusion of *Emiliania huxleyi* SELENOM may be related to the toxicity of algal blooms [54].

The fusion of two selenoprotein genes, GST (glutathione S-transferase) and MSRA (methionine sulfoxide reductase A), was found in *E. huxleyi*. The structure of the fusion gene is composed of 4 exons, which is also indicated by the EST sequences (Fig. 4d). Multiple sequence alignment of this fusion protein and other selenoproteins shows its homology (shown in family page of Selenoprotein Database). This is the first study to identify a fusion event involving two selenoprotein genes. GST participates in the detoxification of reactive electrophilic compounds by catalyzing their conjugation to glutathione. MSRA reverses the inactivation of many proteins due to the oxidation of critical methionine residues by reducing methionine sulfoxide (MetO) to methionine. GST and MSRA are both considered detoxification enzymes because of their antioxidant function. It has been reported that GST and MSRA were coinduced during chemical stress conditions in bacteria [55, 56], suggesting the correlation of their function and biological processes. This protein fusion in *Emiliania huxleyi* involves the enhancement of the association of these two related genes. Further efforts are needed to explore the biological pathways involving these two enzymes.

## Discussion

**Fig. 5:**
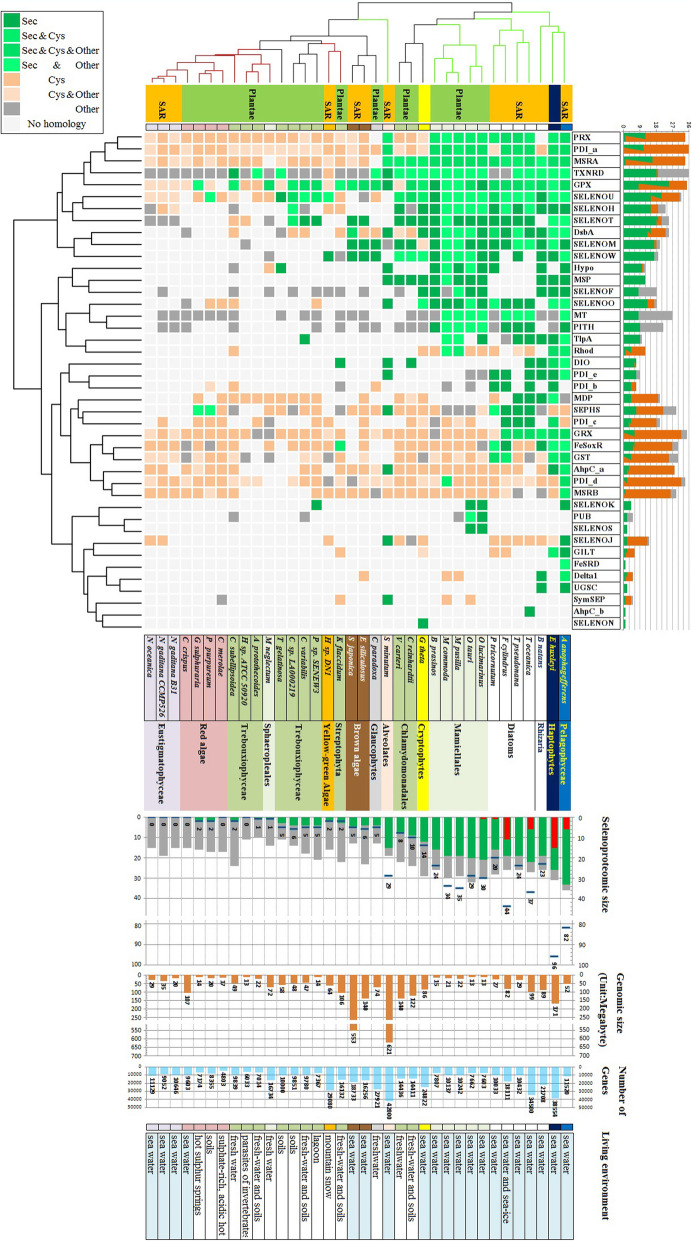
Heatmap of algae selenoprotein distribution. The selenoprotein families and organisms were clustered based on the existence of selenoproteins or different types of homologies. The cluster trees are shown on the top and left side of the heatmap. In the organism cluster tree, the green/red branches indicate high−/low-level selenium algae, which is also shown in Fig. 1. The colored cells with different shades in the heatmap indicate the existence of the different types of selenoproteins or homologies. The meaning of the colors is shown in the top-left corner square. For example, “dark green”, labeled with “Sec” indicates the exclusive existence of selenoprotein; “light green”, labeled with “Sec & Cys”, indicates that selenoprotein and Cys-containing homology were both identified; “gray”, labeled with “other”, indicates the exclusive existence of homologs containing neither Sec nor Cys. The taxonomic description of algae, such as Plante, SAR group, Diatoms, Red algae, etc., is shown beside the organism names with different color backgrounds. On the bottom, the selenoproteome size, genomic size, gene numbers, and living environments of each organism are shown in order. In the chart of “selenoproteome size”, the length of the whole column (composed of green and gray areas) represents the total number of protein families (including selenoproteins and other homologies) of each species. The length of the green bar indicates the number of selenoprotein families. Additionally, the red bar inside the column indicates the number of genomic flanking region duplications found in a specific organism

The history of eukaryotic algae (approximately 1.5 billion years of evolution) is much longer than that of metazoans, so the diversity of the composition and scale of algal selenoproteomes is also higher than that of multicellular metazoan selenoproteomes. In this study, we found a total of 42 selenoprotein families in eukaryotic algae. Nineteen of them have also been reported in animals, such as AhpC, DsbA, MSRA, SPS, GPX, DIO, TNXRD, SELENOF, SELENOH, SELENOJ, SELENOK, SELENOM, SELENON, SELENOO, MSRB, SELENOS, SELENOT, SELENOU, and SELENOW [20]. Interestingly, SELENOJ, which was initially thought to only exist in animals, was also found in algae [37]. It can be seen that most animal selenoproteins have common ancestors with algae homologs. Only a very few selenoproteins are unique to multicellular organisms, such as SELENOE, SELENOI, SELENOP, and SELENOV [19, 57]. To more clearly show the differences in the distribution of selenoprotein families in different algal evolutionary branches, we clustered algal selenoprotein and homolog data, as shown in Fig. 5. As seen from the top cluster tree of Fig. 5, PRX, PDI_a, MSRA, TXNRD, GPX, SELENOU, SELENOH, SELENOT, DSBA, SELENOM, and SELENOW are the most widely distributed Sec-containing proteins in algae. In contrast, SELENOK, PUB, SELENOS, SELENOJ, GILT, FeSRD, Delta1, UGSC, SymSEP, AhpC_b, and SELENON are the least widely distributed Sec-containing proteins.

On the other hand, the cluster tree on the uppermost side of Fig. 5 shows the selenoprotein composition pattern of different algae. The species containing the most Sec-containing family members are clustered on the right side of Fig. 5, and the species with fewer or no selenoproteins are concentrated on the left side. In general, *Aureococcus anophagefferens* and *Emiliania huxleyi* have the most selenoprotein families and the most selenoprotein genes. The composition of the *Bigelowiella natans* selenoproteome is similar. The number of selenoproteins in the four diatom and five Mamiellales species was also higher. The above species can be classified as high-level selenium utilization groups and are marked by green branches in Fig. 5. Red algae and Eustigmatophyceae have the least number of selenoprotein families and are marked by red branches. Other species have medium or small selenoproteomes.

In Fig. 5, the species in the same evolutionary branch (such as microalgae, diatoms, red algae, and Eustigmatophyceae) are also clustered in the same subtree due to the similarity of selenoproteome composition patterns. It is implied that selenium utilization in living organisms has strong cross-species conservation in a particular evolutionary differentiation period. This conservation does not seem to be influenced by other factors. For example, the living environments of red algae are very diverse, including seawater, soils, and hot acidic springs. However, the composition of selenoproteins and their homologs are similar. Additionally, various algae can survive at low temperatures, such as *Fragilariopsis cylindrus*, *Heterococcus sp. DN1* f, and *Coccomyxa subellipsoidea C-169* [58, 59]. Similar cryogenic environments do not lead to similar selenoprotein composition patterns. In fact, selenoproteins are scattered among the high-, medium-, and low-level groups in Fig. 5. The selenoproteomes of these species are more similar to those of evolutionarily closely related species.

Advanced multicellular plants have been reported to completely lose selenoproteins [21]. Is multicellularization related to the loss of selenoproteins? The results of this paper do not reveal a direct relationship between them. Multicellularization occurs independently in several different evolutionary branches, such as *Ectocarpus siliculosus* and *Saccharina japonica* in the SAR group, *Chondrus crispus* in red algae of the Plantae group, *Volvox carteri* in Chlorophyta, and *Klebsormidium flaccidum* in Streptophyta [60–62]. Their selenoproteomes did not significantly change compared to those of closely related unicellular species. For example, the unicellular algae *Chlamydomonas reinhardtii*, belonging to Chlamydomonadales, has a selenoproteome similar to that of *Volvox carteri.* Another example is *Chondrus crispus*, which has the smallest selenoproteome, similar to that of other unicellular red algae. *Klebsormidium flaccidum* and Embryophyta belong to Streptophyta, but they belong to different branches. *Klebsormidium flaccidum* belongs to Klebsormidiophyceae, and Embryophyta belongs to Streptophytina. We also identified the selenoprotein genes of *Nitella hyalina* and *Chaetosphaeridium globosum* belonging to Streptophytina (predicted only from Est data). *Klebsormidium flaccidum* (land organism) and *Nitella hyalina* (freshwater organism) are multicellular algae, while *Chaetosphaeridium globosum* is a unicellular alga. *Klebsormidium flaccidum* has a smaller selenoproteome, while *Nitella hyalina* and *Chaetosphaeridium globosum*, which are more similar to terrestrial plants, have a larger selenoproteome. In summary, it can be inferred that multicellularization occurred independently in multiple evolutionary intervals, and no correlation was found between multicellularization and the reduction of the size of selenoproteomes. The complete loss of selenoproteins in higher plants is likely to be unrelated to the multicellularization of plants.

The aquatic or terrestrial living environment is an essential factor affecting the size of the selenoproteome in multicellular eukaryotes [21]. However, there is not enough evidence in algae to demonstrate the effect of this factor. As shown in Fig. 5, seven algae live in a terrestrial environment. Four (*Chlamydomonas reinhardtii*, *Klebsormidium flaccidum*, *Coccomyxa sp. LA000219*, and *Auxenochlorella protothecoides*) live in both freshwater and soil, and the other three (*Coccomyxa sp. LA000219*, *Trebouxia gelatinosa*, and *Porphyridium purpureum*) are found only in soil. Their selenoproteome sizes range from 1 to 10. The trend of the reduction of selenoproteome size due to adaptation to terrestrial life has not been revealed. In contrast, the selenoproteomes of terrestrial algae are more similar to those of purely aquatic algae within a common evolutionary embranchment.

However, habitat change from seawater to freshwater (including land) of algal ancestors seems potentially to be a critical factor in reducing the size of the selenoproteome. As shown in Fig. 5, all algae in the high-level selenium group live in seawater. None of the algae living in nonseawater environments (freshwater, soil, lagoons, or hot springs) are in the high-level groups. We speculate that the ancestors of eukaryotes with richer selenoproteomes live in the sea. After differentiating into various branches, such as SAR and Plantae, both experienced the process of separation from seawater separately. In this process, some selenoprotein genes and other genes, such as selenoprotein synthesis genes, have been lost. In Plantae, most of the red algae, which have the least number of selenoproteins, live in nonseawater environments, such as hot springs or terrestrial habitats. However, one of them, *Chondrus crispus*, lives in seawater. Similar to those of other red algae, its ancestors experienced the loss of selenoproteins caused by separation from seawater. Without a functional synthetic system, no selenoprotein production could be easily regained even after returning to seawater. In the SAR group, Nannochloropsis, which lost its selenoproteins, is a branch of Eustigmatophyceae. Additionally, the vast majority of Eustigmatophyceae live in freshwater. Nannochloropsis is one of the rare groups that live in seawater. We speculate that the ancestors of Nannochloropsis also experienced a process by which they first migrated to nonseawater and then returned to the sea, which caused the loss of selenoproteins.

Additionally, parasitism is another potential factor thought to be related to the selenium level of specific species. *Helicosporidium sp. ATCC 50920,* which is one of the Trebouxiophyceae, is the only green alga found to have lost selenoproteins entirely. It is a parasite that has been surprisingly recently discovered as a green alga in invertebrates. Its evolution into parasitic life occurred only in the last 100 million years. It was reported that because of its parasitic lifestyle, its genome has also decreased in size. Some genes, such as photosynthesis-related genes, have been lost. We speculate that parasitic lifestyles could cause the loss of selenoprotein genes in *Helicosporidium sp. ATCC 50920*. The parasitic-induced loss of selenoproteins was found and discussed in relation to multicellular eukaryotic organisms in our earlier work. In three representative Platyhelminthes, *Schmidtea mediterranea, Schistosoma japonicum,* and *Taenia solium*, along with the increase in parasitism, the number of selenoprotein genes decreased significantly in the genome [63]. The parasite-induced loss of selenoproteins can also be found in other single-celled eukaryotic organisms besides green algae, such as Plasmodium. Plasmodium and the symbiotic alga *Symbiodinium minutum* both belong to the alveolates. The number of selenoproteins in parasitic Plasmodium is reported to be less than 4, and in most Plasmodium, no selenoproteins are found [64]. However, in the genome of *Symbiodinium minutum,* we detected 11 selenoprotein families that include 26 selenoprotein genes. Moreover, its genome is not complete, so the real selenoproteome may be larger than we observed. Therefore, parasitic life is potentially an important cause of the reduction of the size or even the total loss of the selenoproteome. On the other hand, symbiosis seems to have no impact on selenoproteome composition and size compared to parasitism. The symbiotic algae in this paper include the marine symbiotic alga *Symbiodinium minutum* (symbiotic with coral polyps) and the terrestrial symbiotic alga *Coccomyxa sp. LA000219* and *Trebouxia gelatinosa* (symbiotic with fungi, forming lichens) [58, 65, 66]. These symbiotic algae are all autotrophic organisms, and their survival does not depend on commensal species (polyps or fungi). Therefore, the size of the genome and the number of coding genes are not significantly reduced. Correspondingly, there was no significant change in the number of selenoproteins.

Multiple gene copies of selenoproteins were found in some algae. For example, ten GPX genes were found in the genome of *Emiliania huxleyi*. Eight of them formed four pairs of “highly similar copies”, in which the percentage of positive substitutions between each other was greater than 98% (shown in Figure S9). We analyzed the similarity between all members of each selenoprotein family in each alga. Furthermore, to show the genomic level similarity, the flanking regions of these genes were also compared. All of the selenoprotein gene-flanking genomic level similarity events found in algae are shown in Fig. 5 and Figure S9E. It should be noted that the highest number of genomic level similarity events were found in the four species (15 in *E. huxleyi*, 6 in *Aureococcus anophagefferens*, 11 in *Fragilariopsis cylindrus,* and 6 in *T. oceanica*) with the highest number of selenoprotein genes. Among these species, not only the number of selenoprotein genes but also the total number of coding genes is large. The number of genes in *Emiliania huxleyi* is more than 38,000, and the number of genes in two diatoms (*Fragilariopsis cylindrus* and *Thalassiosira oceanica*) is approximately twice that of the other two diatoms.

Genomic recombination, replication, and transposition are essential events related to the evolution and differentiation of homologous genes and the generation of new genes. The gene-level and genomic level similarity between the various members of the selenoprotein family of the same species reflects the differentiation processes of these gene families. The multiple genomic-level replication events found in the four species with the most selenoprotein genes explain why they have a large number of selenoprotein genes and a large number of selenoprotein families. On the other hand, more frequent recombination and transposition events will lead to increased gene clustering, gene fusion, and even formation of pseudogenes, which is consistent with our previous results (Fig. 4). Interestingly, the algae with the highest number of selenoprotein genes have shown strong environmental adaptability. For example, *Emiliania huxleyi* and *Aureococcus anophagefferens* are widely distributed algae with strong environmental adaptability (they can grow at a wide range of temperatures and have a wide geographical distribution and low light requirements) [67]. *Fragilariopsis cylindrus* is found in seawater and sea ice in the polar ocean and has cold resistance. In addition, the above three algae can form large algal blooms. Their strong environmental resilience and their ability to form algal blooms are generally considered to be related to the amplification of genomes and may also be related to their large numbers of selenoprotein genes.

## Conclusion

In this paper, the following conclusions were drawn from the prediction and comparative analysis of the selenoprotein genes of eukaryotic algae. The systematic distribution of eukaryotic algae selenoproteins was determined, and the first algae selenoprotein database were built. The distribution shows that the ancestors of eukaryotes may have more abundant and more comprehensive selenoproteomes. A potentially critical factor involved in reducing the selenoproteome is the habitat change of algal ancestors from seawater to freshwater or land. Another factor involved in selenoproteome reduction is parasitism. We found three novel selenoprotein families, PDI_e, AhpC_b, and SymSEP. Notably, we first discovered the consecutive Sec-Sec motif (in PDI_e) in selenoproteins. We also described the Sec substitution patterns, gene clustering, and gene fusion events of algae, including the identification of the first two selenoprotein fusion genes (GST and MSRA).

The systematic identification and research of algae selenoprotein genes revealed the primordial state of the eukaryotic selenoproteome. It is important to determine the origin of selenoprotein genes and answer the ultimate question regarding the significance of selenium and selenoproteins to life. Moreover, this is also an indispensable and integral part of revealing the evolutionary spectrum of selenoproteins in all life on earth.

## Methods

### Data resources

The genomic and/or EST sequence data from 137 algal species were downloaded from the current Entrez Genome Project at NCBI; 36 of these species have genome sequences. The accession number, size of the fasta files, and the numbers of sequences of each species are shown in Table S1 (genomic data) and Figure S1 (EST data) in supplementary file 1.

### Prediction of selenoproteins and other homologous genes in algae

The genome and EST sequences were scanned by the SelGenAmic-based algorithm we developed previously. Then, all the open reading frame sequences, including in-frame TGAs, were predicted and searched by BLAST (2.2.25) against a database composed of a known selenoprotein database (collected from our previous work and other selenoprotein studies) and the NR database to find hits with conserved local regions flanking Sec-TGA. The predicted Est sequences were compared to the genome sequences with Splign [68]. It is helpful to recheck the exon-intron splice gene structures of predicted selenoprotein genes. The SECIS elements were searched for by SECISearch online in the sequences downstream of all selenoprotein genes. After all of the selenoprotein genes were identified, all of the homologous genes (Cys-containing and other non-Sec/Cys homologs) were predicted with BLAST and Prosplign from the algae sequences. All of the indels (insertions or deletions), which cause frameshifts, were found in the Prosplign results. Those genes with in-frame indels predicted from genome sequences (without EST alignment evidence) were considered pseudogenes.

The classification and nomenclature used for each selenoprotein family are mainly based on the above described similarity comparison results. The predicted proteins were preclassified and named based on the best hit information collected from reported works or databases (known selenoprotein databases and the NR database). Then, multiple sequence alignment and phylogenetic analysis were carried out to determine the subfamily relationship. If no known family name could be found in the information from the best hits, then conserved domain information was used to name the predicted selenoprotein families. SymSep was named in a different way than the other selenoproteins. Because it is only found in *Symbiodinium phyla*, it was named the Symbiodinium selenoprotein (abbreviated as SymSep).

### Conserved domains and gene cluster analysis

Multiple alignment programs, such as Muscle (3.8.31), were used for the analysis of each selenoprotein family [69]. The phylogenetic tree of each selenoprotein family was built by MrBayes and drawn by FigTree (v1.4.4) [70, 71]. The program InterProScan was used to find all conserved domains and the active site information for each selenoprotein amino acid sequence [72]. BioPerl models such as Bio::Graphics were used to determine the location of the conserved domains for each family (shown in family page of Selenoprotein Database) [73]. In this way, all the fused genes with a new domain could be found. The genomic locations of all algae selenoprotein genes and homologous genes were analyzed by Perl programs. All the gene clusters composed of selenoprotein homolog genes are summarized in Figs. 2 and 4a, and Figure S8.

### Genomic duplication and synteny analysis

Similarity alignment was performed between each selenoprotein pair of the same species from the same family using bl2seq. If the blast positive rate exceeded 50%, the similarity between the flanking genomic sequences of the selenoprotein genes was further analyzed. The DNA sequence of 10,000 bp in length on both sides of the selenoprotein gene was used to build flanking genomic segments for comparison. If the length of the similar region (blast identity rate > = 80%) of those genomic segments exceeded 40,000 bp (20% of 200,000 bp), it was considered to represent genomic duplication. The summary of the genomic duplications flanking all of the selenoprotein genes in algae is shown in Figure S9. For the genomic regions with genomic duplication or genomic level similarity, Mauve (v20150226) was used to calculate and demonstrate the synteny between genomes [74].

### Database of algal selenoproteomes

The algal selenoprotein database website is based on the LAMP framework, runs on the open-source software Apache 2.4.37 and is written in HTML and PHP. The main layout of the web page is built with HTML. The connection of the web page and database and the back-end database management is implemented by PHP. The selenoprotein data are stored in the relational database based on Mysql5.7. The database is created, deleted, updated, and backed up by the MySQL workbench software.

## Supplementary information

**Additional file 1: Table S1.** Genome sequences of eukaryotic algae. **Figure S1.** Selenoproteins and their Cys containing homologs of eukaryotic algae. **Figure S2.** Selenoproteins family of eukaryotic algae. **Figure S3.** Phylogenetic trees and multiple alignments of PDI_a, PDI_b, PDI_c, PDI_d, and PDI_e. **Figure S4.** EST evidence of PDI_e and SECIS elements of novel algal selenoprotein. **Figure S5.** Cys homolog of *Symbiodinium minutum* PRX. **Figure S6.** Substitution of Sec with other amino acids in algal selenoproteins. **Figure S7.** Multiple alignments of SELENOW. **Figure S8.** Location of selenoprotein gene clusters in Algae genomes. **Figure S9.** Similarity comparison of *Emiliania huxleyi* GPX selenoproteins and genomic level similarity events found for algae selenoprotein genes. **Figure S10.** Algae selenoprotein database web site. **Figure S11.** Keyword search page. **Figure S12.** Selenoprotein family list search page. **Figure S13.** Selenoprotein detailed information page. **Figure S14.** Multiple sequence alignments. **Figure S15.** Selenoprotein family statistics in SPDB.

## Data Availability

Data can be requested from the corresponding author. The species data used in the study can be obtained from the NCBI according to the ID. Species ID can be found in Table S1 of Supplementary file 1. The information of selenoproteins presented in this paper can be found on the Selenoproteins Database (www.selenoprotein.com) established by us through species, family, and ID information. Please refer to Figures S10 ~ S14 of Supplementary file 1 for the specific application method. The datasets generated and/or analyzed during the current study are available from the Selenoprotein Database.

## Abbreviations

AhpC: Alkyl hydroperoxide reductase domain-containing protein
Cys: Cysteine
DIO: Deiodinase
DsbA: Disulfide bond-forming protein
SecS: Eukaryotic Sec synthase
eEFSec: Eukaryotic Sec-specific elongation factor
FeSoxR: FeS oxidoreductase
FeSRD: FeS reductase
GRX: Glutaredoxin
GPX: Glutathione peroxidase
GST: Glutathione S-transferase
Hypo: Hypothetical selenoprotein
MSP: Membrane selenoprotein
MSRA: Methionine sulfoxide reductase A
MetO: Methionine sulfoxide
MDP: Methylated-DNA-[protein]-cysteine methyltransferase selenoprotein
MT: Methyltransferase
PSTK: O-phosphoseryl-tRNA [Ser] Sec kinase
PRX: Peroxiredoxin
PITH: Proteasome-interacting thioredoxin domain-containing protein
PDI: Protein disulfide isomerase
PUB: PUB domain-containing protein
Rhod: Rhodanase
SECIS: Sec insertion sequence
SBP2: SECIS binding protein 2
Se: Selenium
Sec: Selenocysteine
SEPHS: Selenophosphate synthetase
SELENOE: Selenoprotein E, fish selenoprotein 15
SELENOF: Selenoprotein F
SELENOH: Selenoprotein H
SELENOI: Selenoprotein I
SELENOJ: Selenoprotein J
SELENOK: Selenoprotein K
SELENOL: Selenoprotein L
SELENOM: Selenoprotein M
SELENON: Selenoprotein N
SELENOO: Selenoprotein O
SELENOP: Selenoprotein P
MSRB: Selenoprotein R
SELENOS: Selenoprotein S
SELENOT: Selenoprotein T
SELENOU: Selenoprotein U
SELENOV: Selenoprotein V
SELENOW: Selenoprotein W
SymSEP: Symbiodinium selenoprotein
TXNRD: Thioredoxin reductase
TlpA: Thioredoxin-like protein
UGSC: UGSC-containing protein
pVHL: Von Hippel-Lindau
