# Supplementary material for: The algal selenoproteomes
Source: BMC Genomics. 2020 Oct 7;21:699. doi: 10.1186/s12864-020-07101-z (PMC7539508; doi:10.1186/s12864-020-07101-z)
Supplement: Supplementary file 1 — Additional file 1: Table S1. Genome sequences of eukaryotic algae. Figure S1. Selenoproteins and their Cys containing homologs of eukaryotic algae. Figure S2. Selenoproteins family of eukaryotic algae. Figure S3. Phylogenetic trees and multiple alignments of PDI_a, PDI_b, PDI_c, PDI_d, and PDI_e. Figure S4. EST evidence of PDI_e and SECIS elements of novel algal selenoprotein. Figure S5. Cys homolog of Symbiodinium minutum PRX. Figure S6. Substitution of Sec with other amino acids in algal selenoproteins. Figure S7. Multiple alignments of SELENOW. Figure S8. Location of selenoprotein gene clusters in Algae genomes. Figure S9. Similarity comparison of Emiliania huxleyi GPX selenoproteins and genomic level similarity events found for algae selenoprotein genes. Figure S10. Algae selenoprotein database web site. Figure S11. Keyword search page. Figure S12. Selenoprotein family list search page. Figure S13. Selenoprotein detailed information page. Figure S14. Multiple sequence alignments. Figure S15. Selenoprotein family statistics in SPDB. [file 12864_2020_7101_MOESM1_ESM.docx]

**Table S1 Genome sequences of eukaryotic algae**

|  |  |  |  | **Size (M Byte)** | | | | **Sequences** | | | **Data type** | | |  | **Genome sequence ids** | | | | | | | | |
| --- | --- | --- | --- | --- | --- | --- | --- | --- | --- | --- | --- | --- | --- | --- | --- | --- | --- | --- | --- | --- | --- | --- | --- |
| [***Aureococcus anophagefferens***](file:///D:\WorK\20170303BMP\基因组信息.xlsx#!A) |  |  |  | 52 | | | 5239 | | | scaffold/contig | | |  | ACJI01000001 | | | - | | | ACJI01005239 | | |  |
| [***Nannochloropsis oceanica***](file:///D:\WorK\20170303BMP\基因组信息.xlsx#!A) |  |  |  | 29 | | | 5851 | | | scaffold/contig | | |  | AEUM01000001 | | | - | | | AEUM01005851 | | |  |
| [***Nannochloropsis gaditana***](file:///D:\WorK\20170303BMP\基因组信息.xlsx#!A) |  |  |  | 20 | | | 21 | | | chromosome | | |  | CM002455 | | | - | | | CM002463 | | |  |
| [***Nannochloropsis gaditana CCMP526***](file:///D:\WorK\20170303BMP\基因组信息.xlsx#!A) |  |  |  | 35 | | | 1883 | | | scaffold/contig | | |  | JH470562 | | | - | | | JH472444 | | |  |
| [***Heterococcus sp. DN1***](file:///D:\WorK\20170303BMP\基因组信息.xlsx#!A) |  |  |  | 64 | | | 21589 | | | scaffold/contig | | |  | AXNI01000001 | | | - | | | AXNI01021589 | | |  |
| [***Saccharina japonica***](file:///D:\WorK\20170303BMP\基因组信息.xlsx#!A) |  |  |  | 553 | | | 13327 | | | scaffold/contig | | |  | JXRI01000001 | | | - | | | JXRI01013327 | | |  |
| [***Ectocarpus siliculosus***](file:///D:\WorK\20170303BMP\基因组信息.xlsx#!A) |  |  |  | 140 | | | 34 | | | chromosome | | |  | FN649726 | | | - | | | FN649759 | | |  |
| [***Thalassiosira oceanica***](file:///D:\WorK\20170303BMP\基因组信息.xlsx#!A) |  |  |  | 99 | | | 50891 | | | scaffold/contig | | |  | AGNL01000001 | | | - | | | AGNL01050891 | | |  |
| [***Thalassiosira pseudonana***](file:///D:\WorK\20170303BMP\基因组信息.xlsx#!A) |  |  |  | 29 | | | 20 | | | Chromosome | | |  | CM000638 | | | - | | | CM000655\| | | |  |
| [***Fragilariopsis cylindrus***](file:///D:\WorK\20170303BMP\基因组信息.xlsx#!A) |  |  |  | 82 | | | 271 | | | scaffold/contig | | |  | Scaffold_1/KV784353 | | | - | | | Scaffold_282/KV784623 | | |  |
| [***Phaeodactylum tricornutum***](file:///D:\WorK\20170303BMP\基因组信息.xlsx#!A) |  |  |  | 27 | | | 33 | | | chromosome | | |  | CM000605 | | | - | | | CM000635 | | |  |
| ***[Symbiodinium minutum](file:///D:\\WorK\\20170303BMP\\基因组信息.xlsx#!A)*** |  | |  |  | 621 | | 21898 | | | scaffold/contig | | |  | DF242851 | | | - | | | DF242864 | | |  |
| [***Bigelowiella natans***](file:///D:\WorK\20170303BMP\基因组信息.xlsx#!A) |  |  |  | 89 | | | | 3740 | | | scaffold/contig | | |  | ADNK01000001 | | | - | | | ADNK01003736 | | |
| [***Guillardia theta***](file:///D:\WorK\20170303BMP\基因组信息.xlsx#!A) |  |  |  | 86 | | | | 5130 | | | scaffold/contig | | |  | AEIE01000001 | | | - | | | AEIE01005126 | | |
| ***[Emiliania huxleyi](file:///D:\\WorK\\20170303BMP\\基因组信息.xlsx#!A)*** |  | |  |  | 171 | | 7795 | | | scaffold/contig | | |  | KB863010 | | | - | | | KB870804 | | |  |
| [***Cyanophora paradoxa***](file:///D:\WorK\20170303BMP\基因组信息.xlsx#!A) |  |  |  | 74 | | | | 60119 | | | scaffold/contig | | |  | Contig1 | | | - | | | Contig60119 | | |
| [***Chondrus crispus***](file:///D:\WorK\20170303BMP\基因组信息.xlsx#!A) |  |  |  | 107 | | | 926 | | | scaffold/contig | | |  | HG001459 | | | - | | | HG002383 | | |  |
| [***Porphyridium purpureum***](file:///D:\WorK\20170303BMP\基因组信息.xlsx#!A) |  |  |  | 20 | | | 3014 | | | scaffold/contig | | |  | AROW01000001 | | | - | | | AROW01003014 | | |  |
| [***Galdieria sulphuraria***](file:///D:\WorK\20170303BMP\基因组信息.xlsx#!A) |  |  |  | 14 | | 433 | | | scaffold/contig | | |  | KB454484 | | | - | | | KB454916 | | |  |  |
| [***Cyanidioschyzon merolae***](file:///D:\WorK\20170303BMP\基因组信息.xlsx#!A) |  |  |  | 17 | | 20 | | | chromosome | | |  | AP006483 | | | - | | | AP006502 | | |  |  |
| [***Klebsormidium flaccidum***](file:///D:\WorK\20170303BMP\基因组信息.xlsx#!A) |  |  |  | 106 | | | | 1814 | | | scaffold/contig | | |  | DF236950 | | | - | | | DF238761 | | |
| [***Monoraphidium neglectum***](file:///D:\WorK\20170303BMP\基因组信息.xlsx#!A) |  |  |  | 72 | | | 6720 | | | scaffold/contig | | |  | KK100223 | | | - | | | KK106940 | | |  |
| [***Volvox carteri***](file:///D:\WorK\20170303BMP\基因组信息.xlsx#!A) |  |  |  | 140 | | | 1251 | | | scaffold/contig | | |  | GL378323 | | | - | | | GL379573 | | |  |
| ***[Chlamydomonas reinhardtii](file:///D:\\WorK\\20170303BMP\\基因组信息.xlsx#!A)*** |  |  |  | 122 | | | 1556 | | | scaffold/contig | | |  | DS496108 | | | - | | | DS497664 | | |  |
| [***Micromonas pusilla CCMP1545***](file:///D:\WorK\20170303BMP\基因组信息.xlsx#!A) |  |  |  | 22 | | | | 23 | | | scaffold/contig | | |  | GG663735 | | | - | | | GG663755 | | |
| ***[Micromonas commoda](file:///D:\\WorK\\20170303BMP\\基因组信息.xlsx#!A)*** |  |  |  | 21 | | | | 19 | | | chromosome | | |  | CP001323 | | | - | | | CP001577 | | |
| ***[Bathycoccus prasinos](file:///D:\\WorK\\20170303BMP\\基因组信息.xlsx#!A)*** |  |  |  | 15 | | | | 21 | | chromosome | | |  | NC_023990 | | | - | | | NC_024008 | | |  |
| [***Ostreococcus lucimarinus***](file:///D:\WorK\20170303BMP\基因组信息.xlsx#!A) |  |  |  | 13 | | | | 21 | | | chromosome | | |  | CP000581 | | | - | | | CP000601 | | |
| [***Ostreococcus tauri***](file:///D:\WorK\20170303BMP\基因组信息.xlsx#!A) |  |  |  | 13 | | | | 20 | | | chromosome | | |  | CAID01000001 | | | - | | | CAID01000020 | | |
| [***Trebouxia gelatinosa***](file:///D:\WorK\20170303BMP\基因组信息.xlsx#!A) |  |  |  | 58 | | | | 108 | | scaffold/contig | | |  | KN805262 | | | - | | | KN805369 | | |  |
| ***[Picochlorum sp. SENEW3](file:///D:\\WorK\\20170303BMP\\基因组信息.xlsx#!A)*** |  |  |  | 14 | | | | 880 | | | scaffold/contig | | |  | JPID01000001 | | | - | | | JPID01000880 | | |
| [***Coccomyxa subellipsoidea***](file:///D:\WorK\20170303BMP\基因组信息.xlsx#!A) |  |  |  | 48 | | | | 56 | | | scaffold/contig | | |  | KN714610 | | | - | | | KN714665 | | |
| ***[Coccomyxa subellipsoidea C-169](file:///D:\\WorK\\20170303BMP\\基因组信息.xlsx#!A)*** |  |  |  | 49 | | | | 29 | | | scaffold/contig | | |  | AGSI01000001 | | | - | | | AGSI01000029 | | |
| ***[Helicosporidium sp. ATCC 50920](file:///D:\\WorK\\20170303BMP\\基因组信息.xlsx#!A)*** |  |  |  | 13 | | | | 5666 | | | scaffold/contig | | |  | AYPS01000001 | | | - | | | AYPS01005666 | | |
| [***Chlorella variabilis***](file:///D:\WorK\20170303BMP\基因组信息.xlsx#!A) |  |  |  | 47 | | | | 414 | | | scaffold/contig | | |  | GL433835 | | | - | | | GL434248 | | |
| ***[Auxenochlorella protothecoides](file:///D:\\WorK\\20170303BMP\\基因组信息.xlsx#!A)*** |  |  |  | 22 | | | | 113 | | | scaffold/contig | | |  | KL662078 | | | - | | | KL662190 | | |


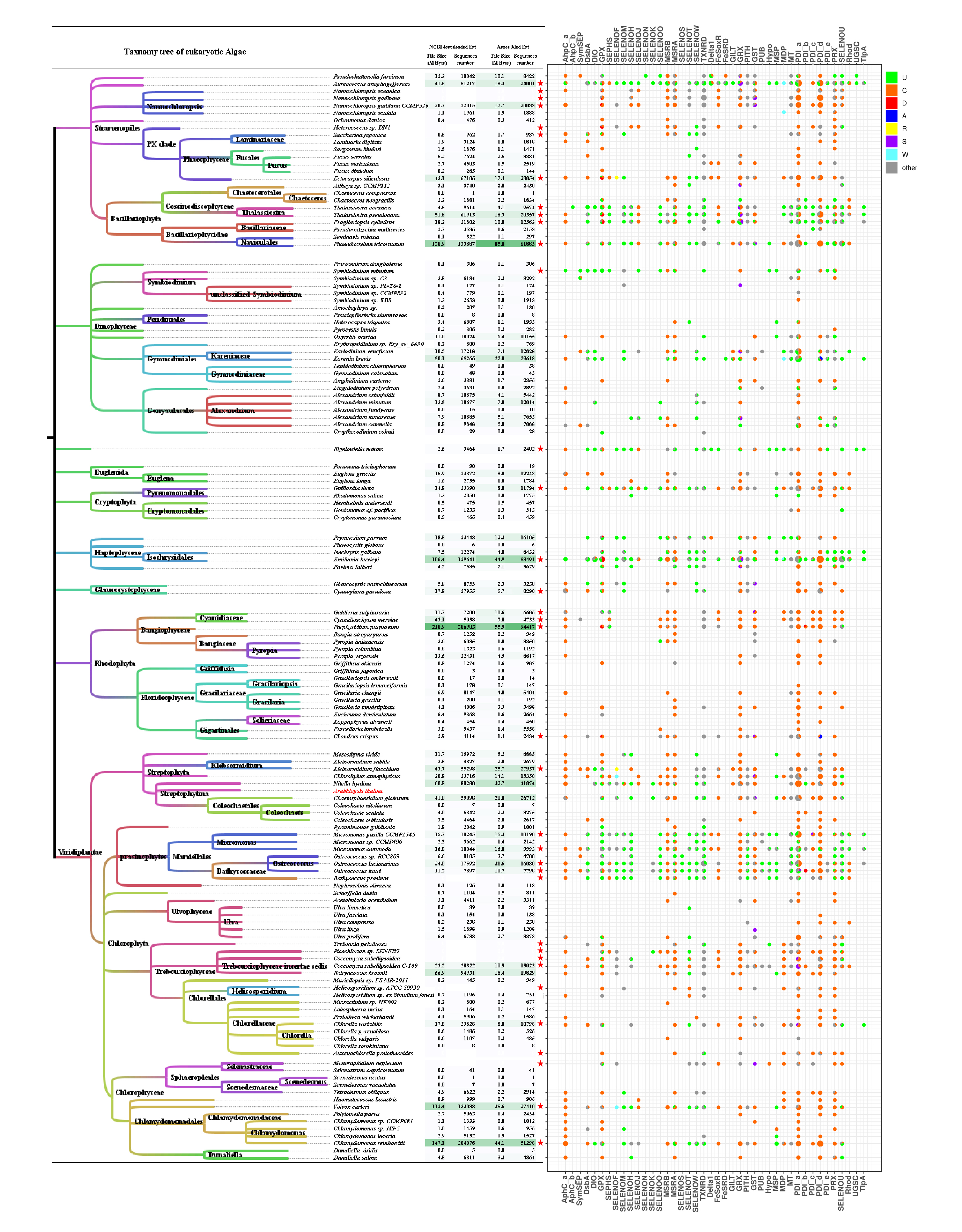


**Figure S1 Selenoproteins and their Cys containing homologs of eukaryotic algae**

All the algae posses genome sequences data are marked with red pentagrams. The data amount of Est and assembled Est contigs of each species are also shown. A pie chart on the right shows the presence or absence of a particular family of selenoproteins in a particular species. The meaning of the color in the pie chart: green indicates true selenoprotein (containing Sec), orange indicates homologous protein (containing Cys), and gray and other colores indicates other homologous proteins (the position corresponding to Sec is neither Sec nor Cys). Different colors represent the type of amino acid at the position corresponding to Sec, and the meaning of the color is shown in the legend on the right. The area of the pie chart and the size of the area of each fan shape symbolically show the number of genes in the corresponding class.


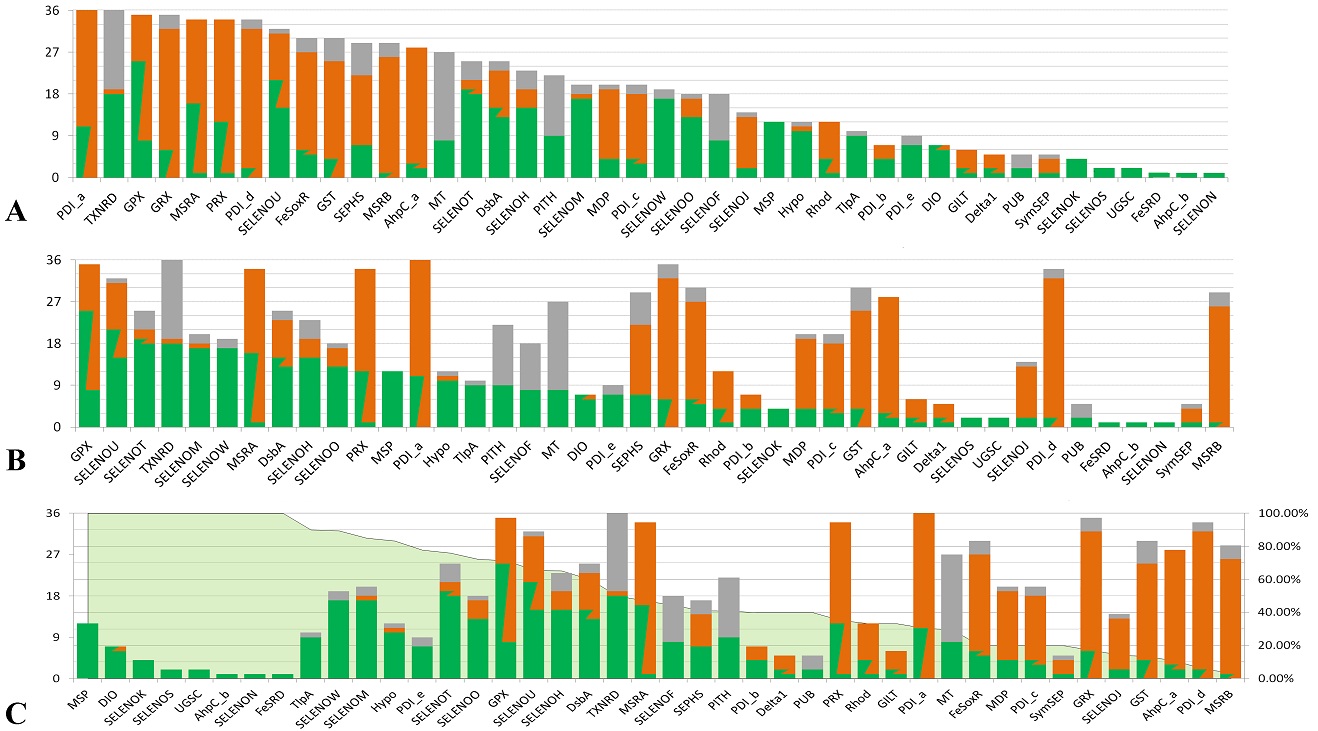


**Figure S2 Selenoproteins family of eukaryotic algae**

The difference in the distribution of selenoprotein families in 36 algae. The color of the column has the same meaning as above. The height of the column represents the number of species containing the protein family. **A.** all selenoprotein families are ranked from high to low according to the total height of the cylinder, **B.** from high to low according to the total height of green part, and **C.** from high to low according to the proportion of Green (Sec).


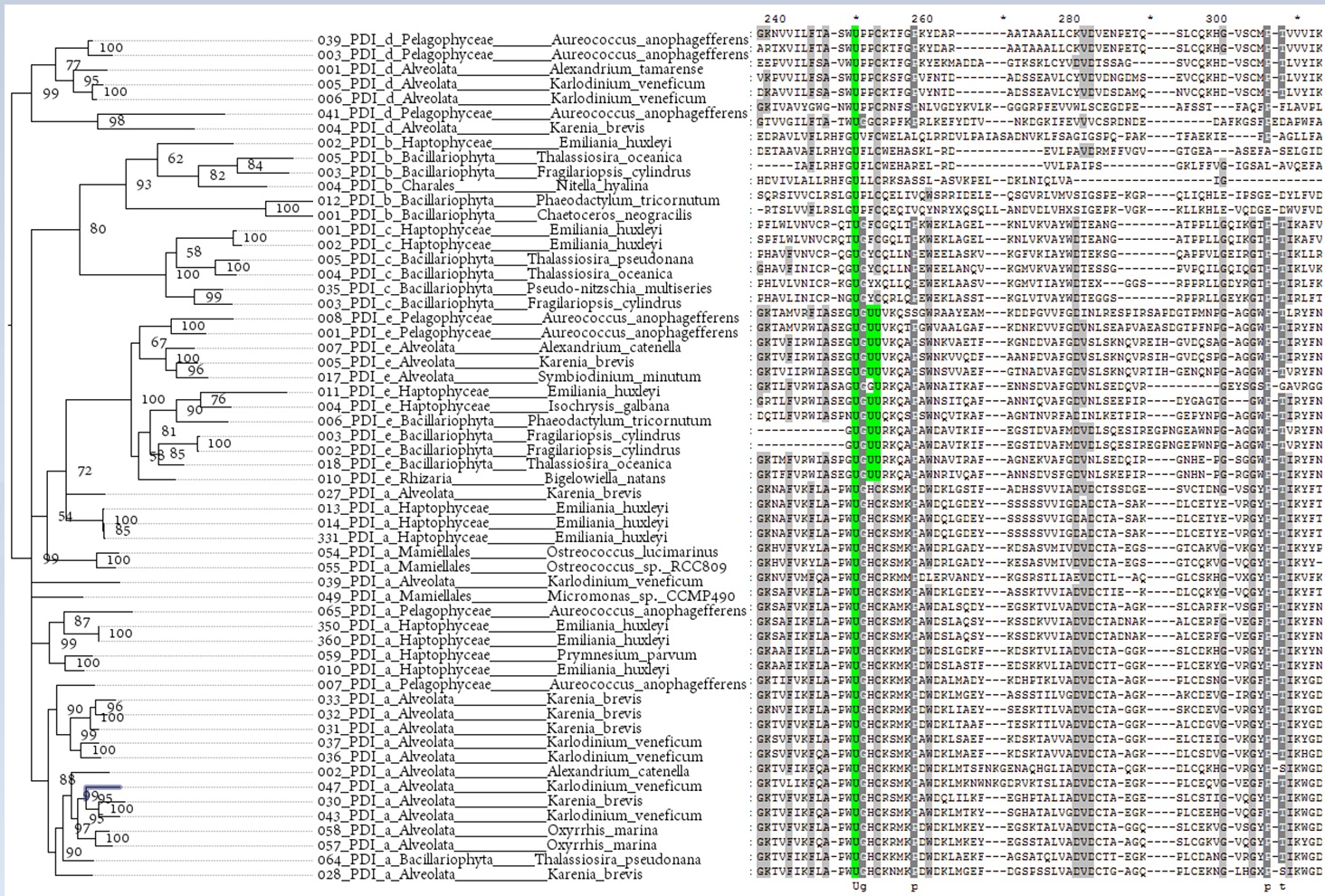


**Figure S3 Phylogenetic trees and multiple alignments of PDI_a, PDI_b, PDI_c, PDI_d, and PDI_e**

The Sec residue is marked with a green background. The sequences numbers, phyla names, and organism names are shown on the left.


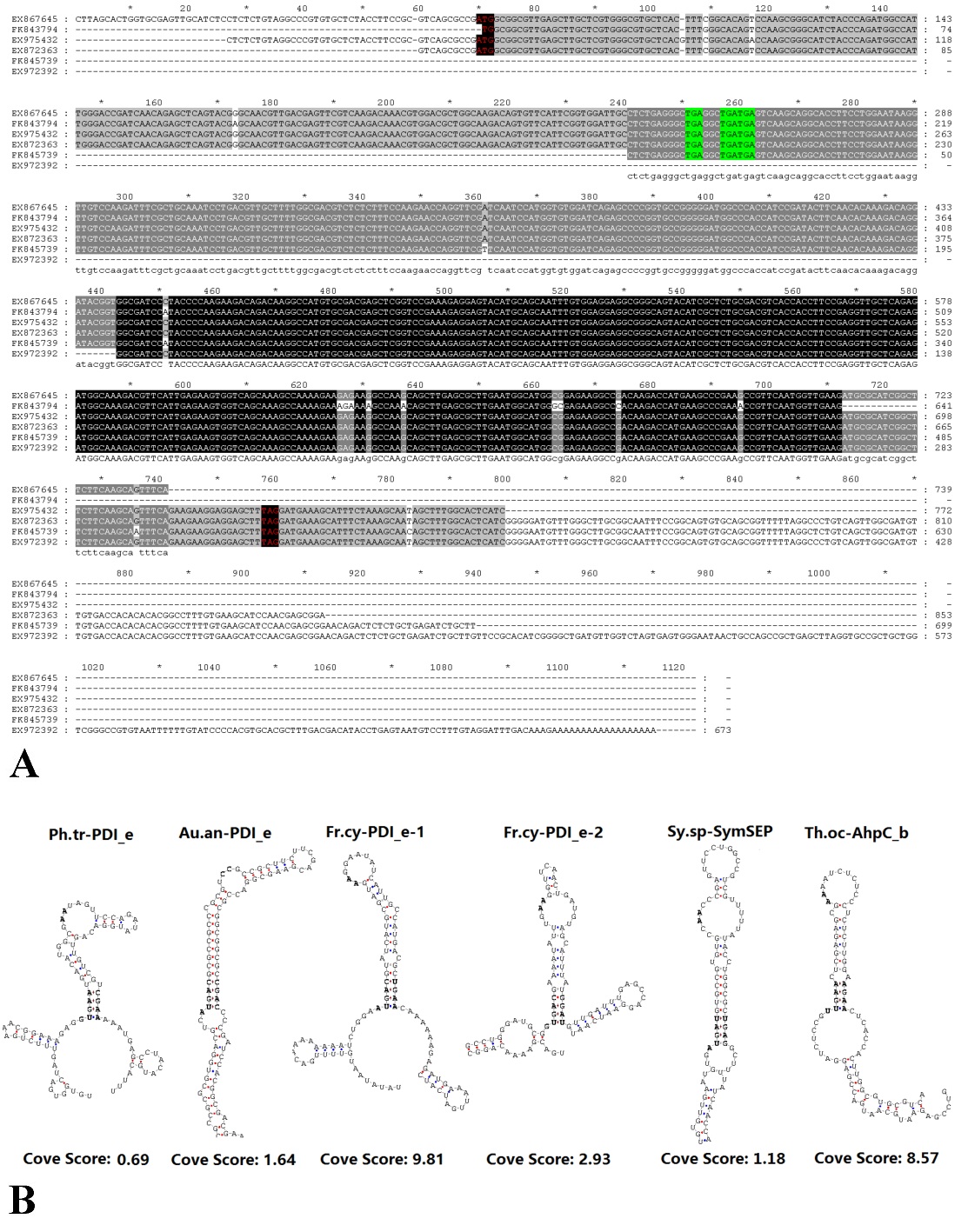


**Figure S4 EST evidence of PDI_e and SECIS elements of novel algal selenoprotein**

**A.** Multiple alignments of PDI_e Ests of *Karenia brevis*, the Sec-TGA codon, the Start and the Stop codon are highlighted. **B.** SECIS elements of PDI_e, Aphc_b, and SymSEP. Abbreviation of organism names: Ph.tr (*Phaeodactylum tricornutum*), Au.an (*Aureococcus anophagefferens*), Fr.cy (*Fragilariopsis cylindrus*), Th.oc (*Thalassiosira oceanica*), Sy.sp (*Symbiodinium sp. C3*)


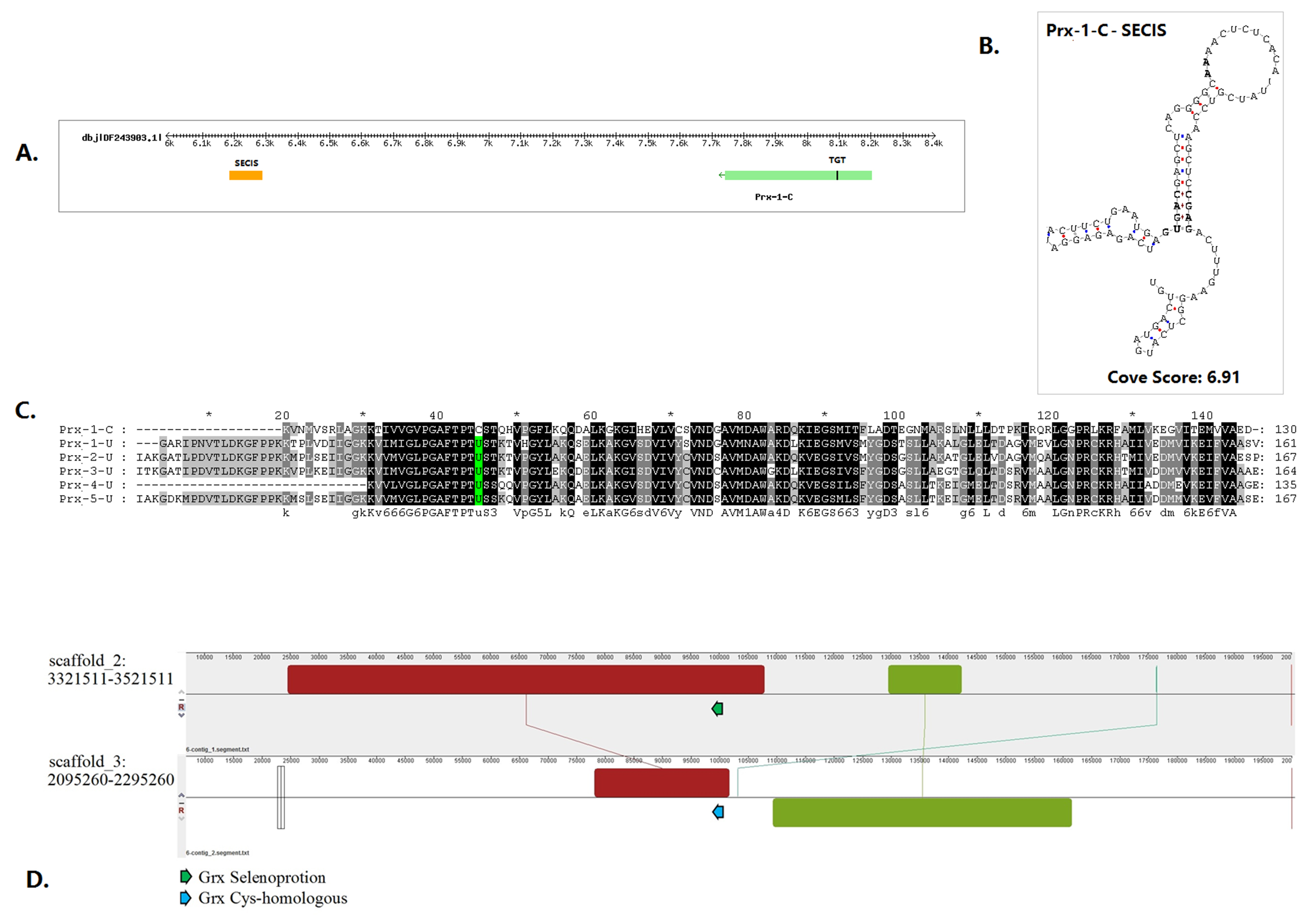
**Figure S5** **Cys homolog of** ***Symbiodinium minutum* PRX**

**A.**The gene structure and genomes position of *Symbiodinium minutum* Prx-1-C. A SECIS element is found downstream. **B.** The secondary structure and Cove Score of SECIS of Sy.mi-Prx-1-C. **C.** Multiple sequences alignment of PRX sequences of *Symbiodinium minutum*. **D.** Genomic synteny of *Fragilariopsis cylindrus* GRX. The left caption and the top ruler indicate the ID and the section of the genome sequence, and colored strip ribbon shows the regions where there is a collinearity relationship between different genomes. The positions of the GRX selenoprotein gene and the Cys homologous gene are shown by the green and blue arrows respectively.


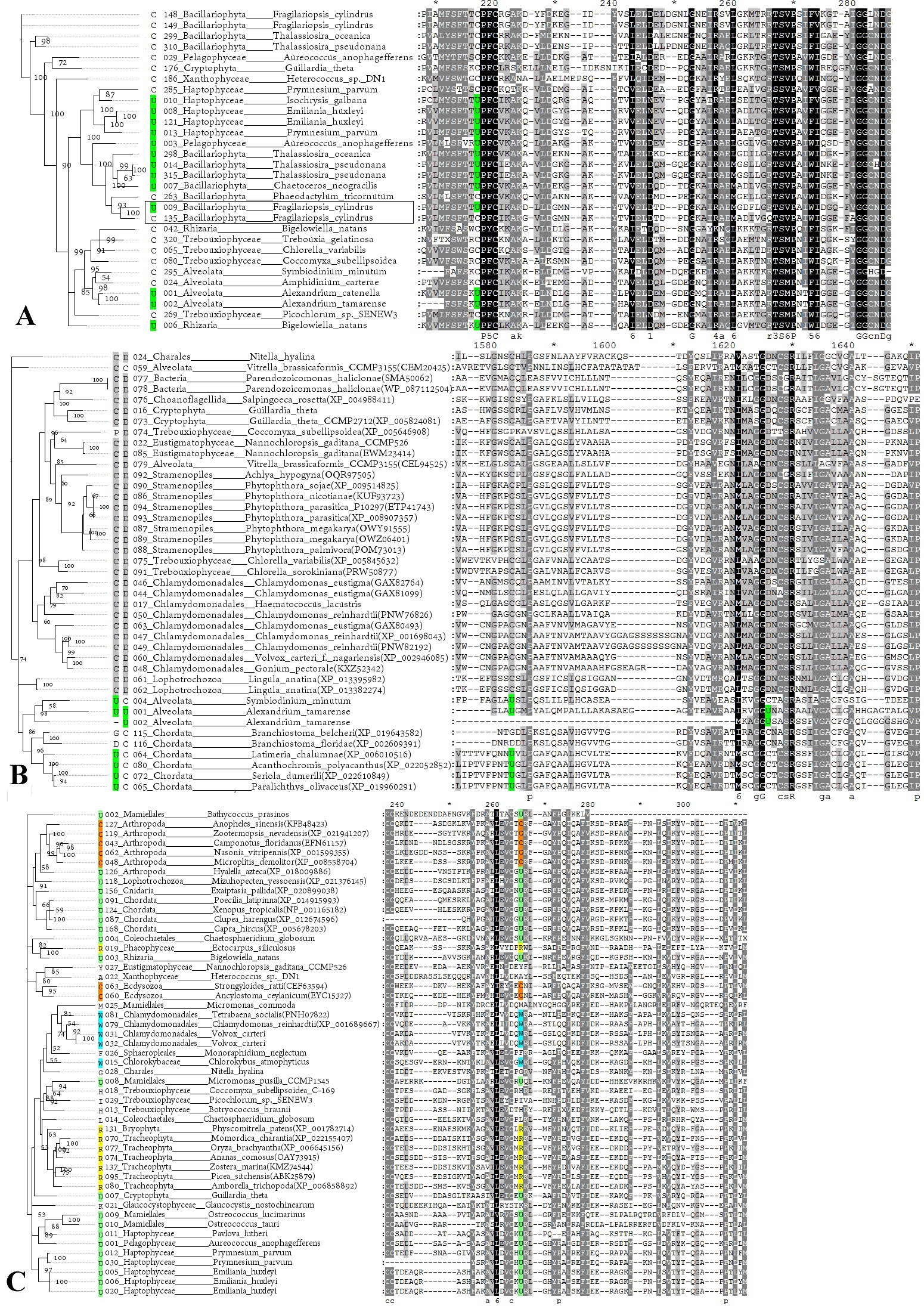


**Figure S6 Substitution of Sec with other amino acids in algal selenoproteins**

Phylogenetic trees and multiple sequence alignments show the Sec substitutions of three selenoprotein families, where U is shown by a green background, and amino acids corresponding to the U position are displayed next to the evolutionary tree. **A.** Examples of Sec->Cys found in GRX of *Fragilariopsis cylindrus*. **B.** Examples of Cys->Sec found in SELENOJ. **C.** Changes in Sec and its motif in SELENOF. The different amino acids at the position corresponding to U are highlighted with a background of different colors.


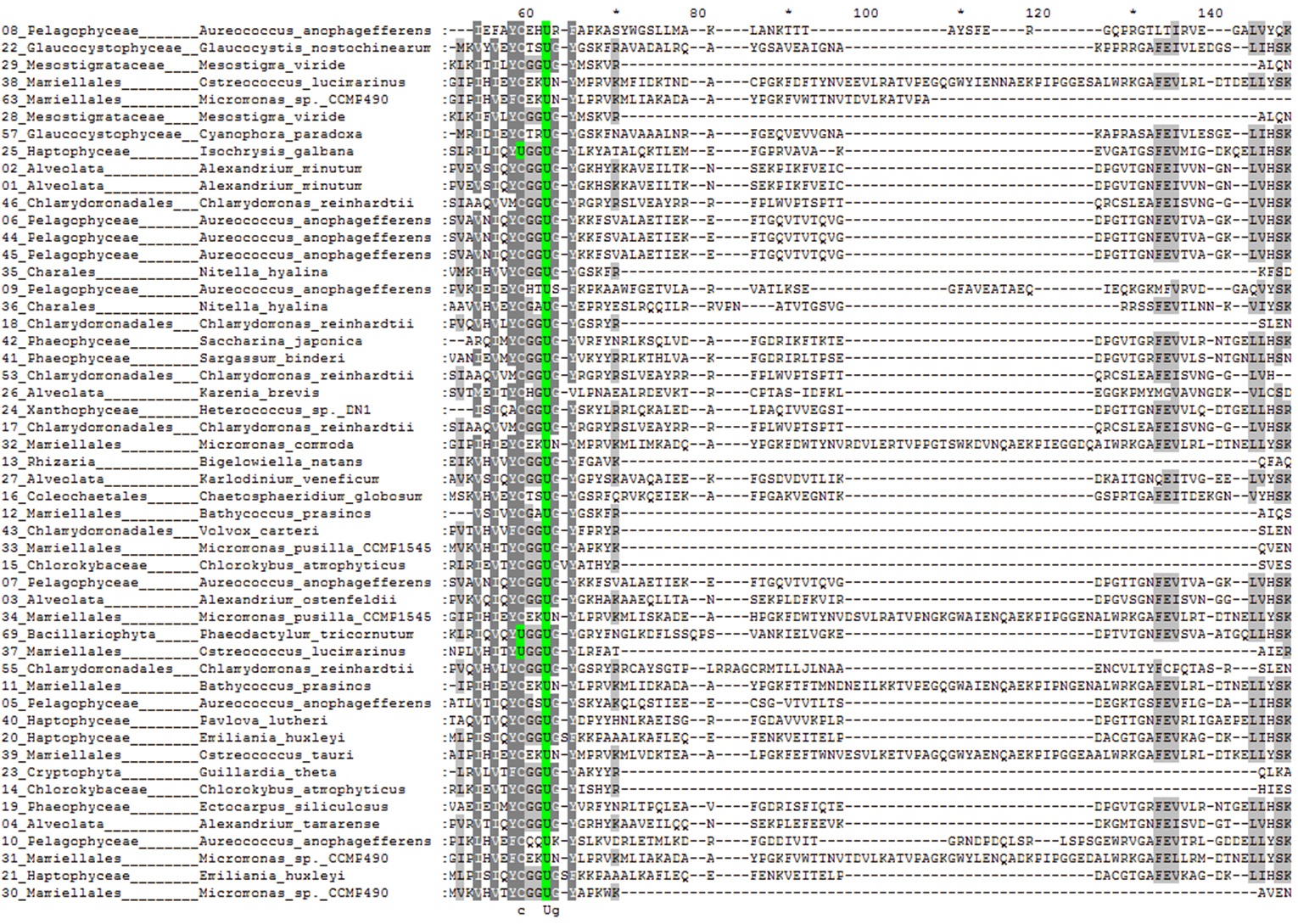


**Figure S7 Multiple alignments of SELENOW**

The Sec residue is marked with a green background. The sequences numbers, phyla names, and organism names are shown on the left.


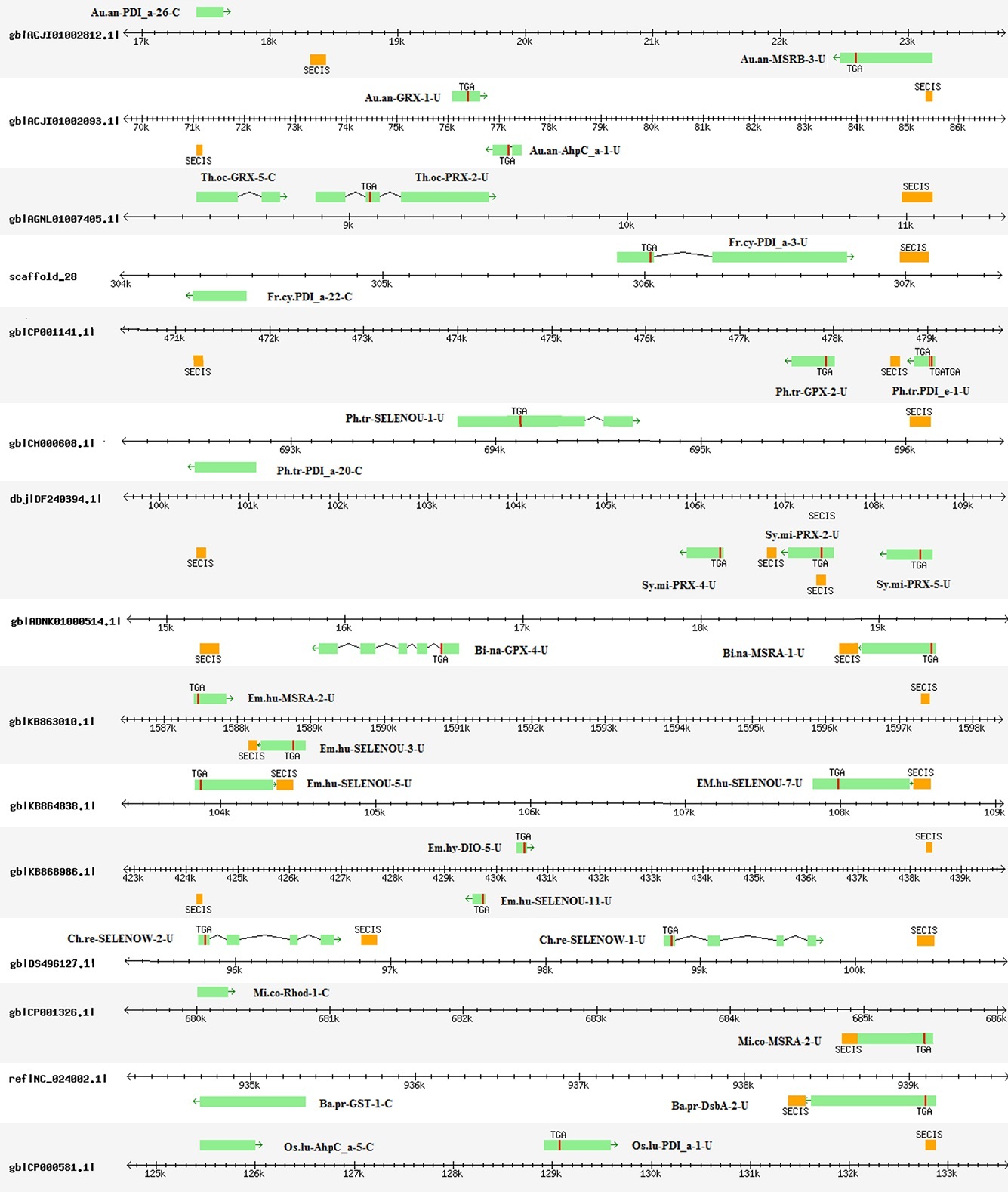


**Figure S8** **Location of selenoprotein gene clusters in Algae genomes**

Abbreviation of organism names: Au.an (*Aureococcus anophagefferens*), Th.oc (*Thalassiosira oceanica*), Fr.cy (*Fragilariopsis cylindrus*), Ph.tr (*Phaeodactylum tricornutum*), Sy.mi (*Symbiodinium minutum*) , Bi.na (*Bigelowiella natans*), Em.hu (*Emiliania huxleyi*), Ch.re (*Chlamydomonas reinhardtii*), Mi.co (*Micromonas commoda*), Ba.pr (*Bathycoccus prasinos*), Os.lu (*Ostreococcus lucimarinus*)


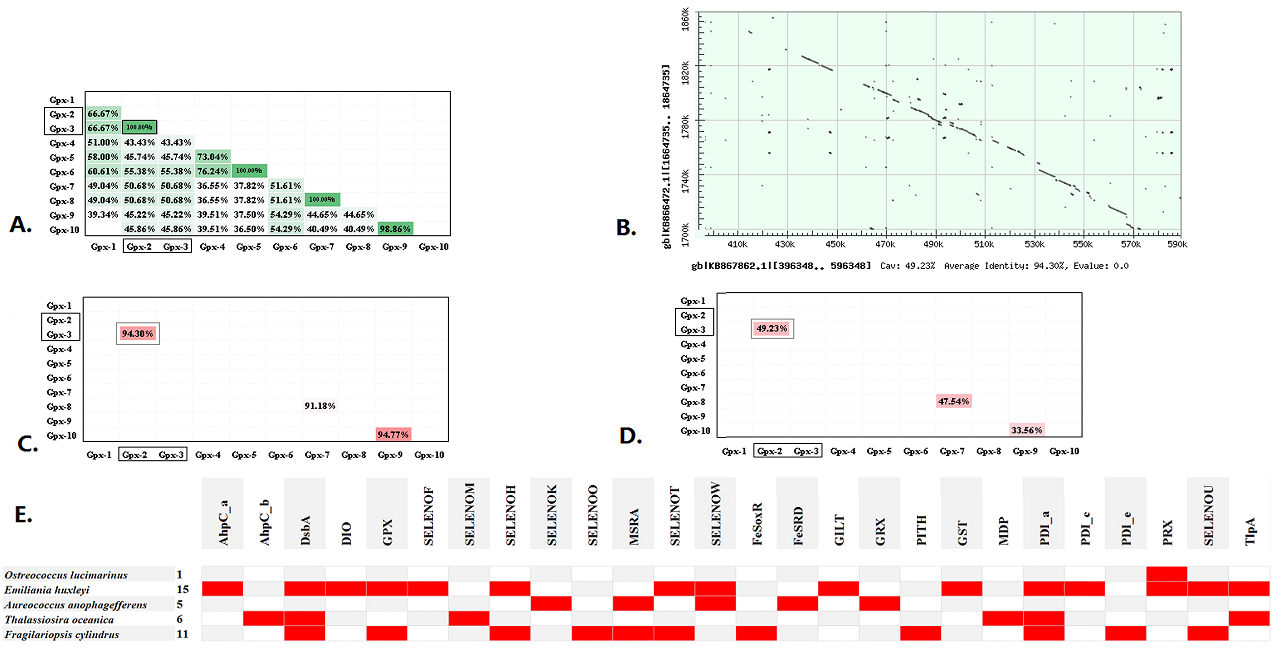


**Figure S9 Similarity comparison of** [***Emiliania huxleyi***](file:///D:\WorK\20170303BMP\基因组信息.xlsx#!A) **GPX selenoproteins and genomic level similarity events found for algae selenoprotein genes**

**A.** The amino acid sequences of those GPX were compared to each other to build the matrix. The percent positive substitutions are shown in the cells of the matrix is extracted from bl2seq results. The deeper the green background, the higher the percent positive substitutions. **B.** The genome sequences (length in 200000 bp) flanking those GPX were compared by bl2seq. For example, the dot plot of similarity between GPX-2 and GPX-3 was shown. The percent sequence identity is 94.38%, and the query genomic coverage is 49.23%. For each pair of GPX genes, the genomic flanking region was compared. Those with high similarity (the percent sequence identity >= 80%, the query coverages >= 20%) were considered as gene pairs with detectable genomic level similarity. **C.** The percent identities of all the genomic level similar GPX pairs are shown. **D.** The query coverages of all the genomic level similar GPX pairs are shown. **E.** The matrix of all the gene pairs with a detectable genomic level similarity of algae.


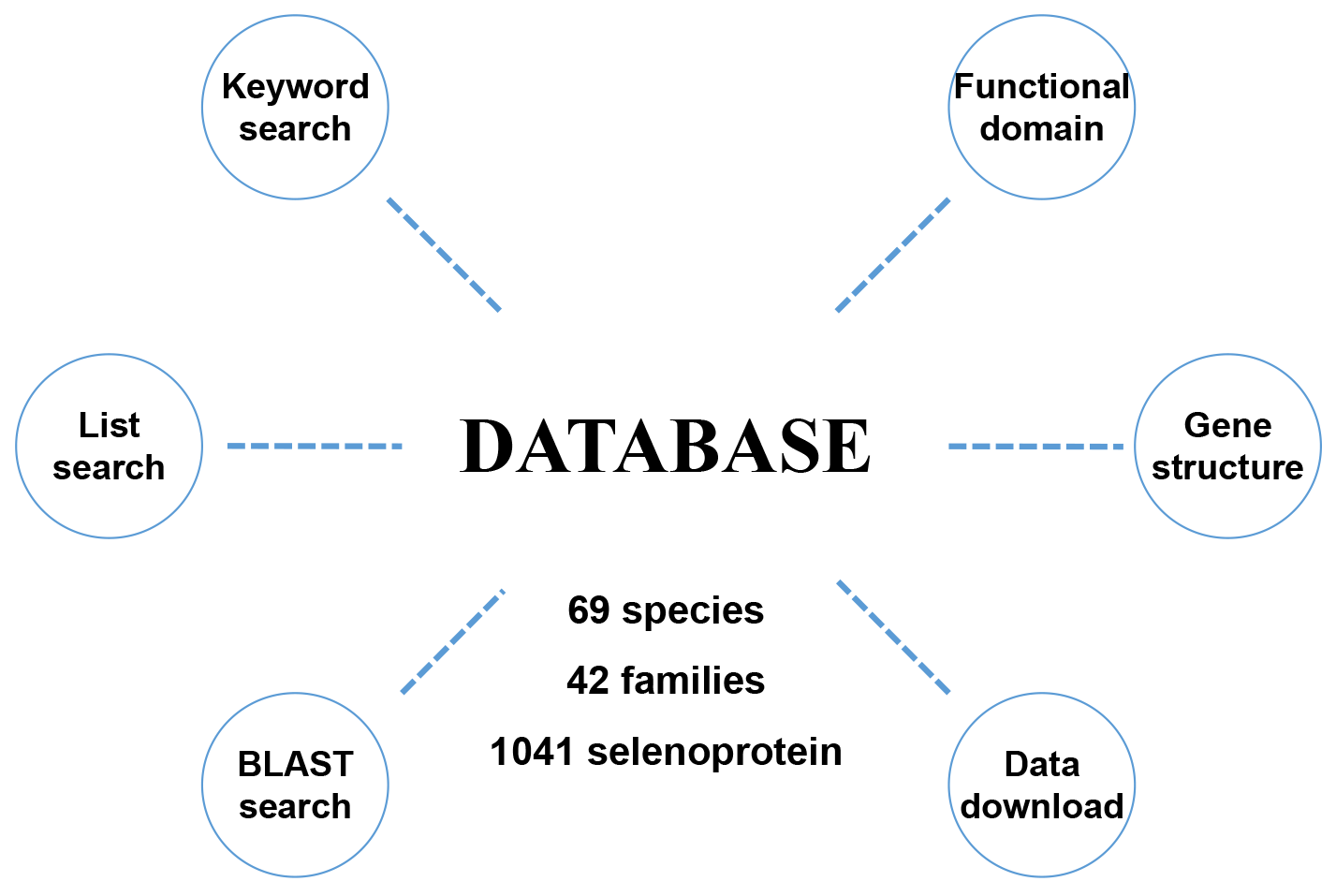


**Figure S10** **Algae selenoprotein database web site**

Currently, the database contains 1041 selenoprotein data of alga. The site is currently open for data search and data download services. In the query service, keyword query can be used for precise search according to protein name, species, protein family, etc. You can also use the species and protein family table provided by the website to search. Blast pages can also be used for Blast alignment lookups by providing nucleic acid or amino acid sequences. The DATA page provides the download of the statistical chart of the website DATA analysis. Each piece of selenoprotein data contains relatively complete data information, including protein name, genome information, EST information, protein sequence, gene sequence, SECIS element sequence, structural domain, species, protein family. All these provide a detailed data basis for future research of selenoprotein researchers.


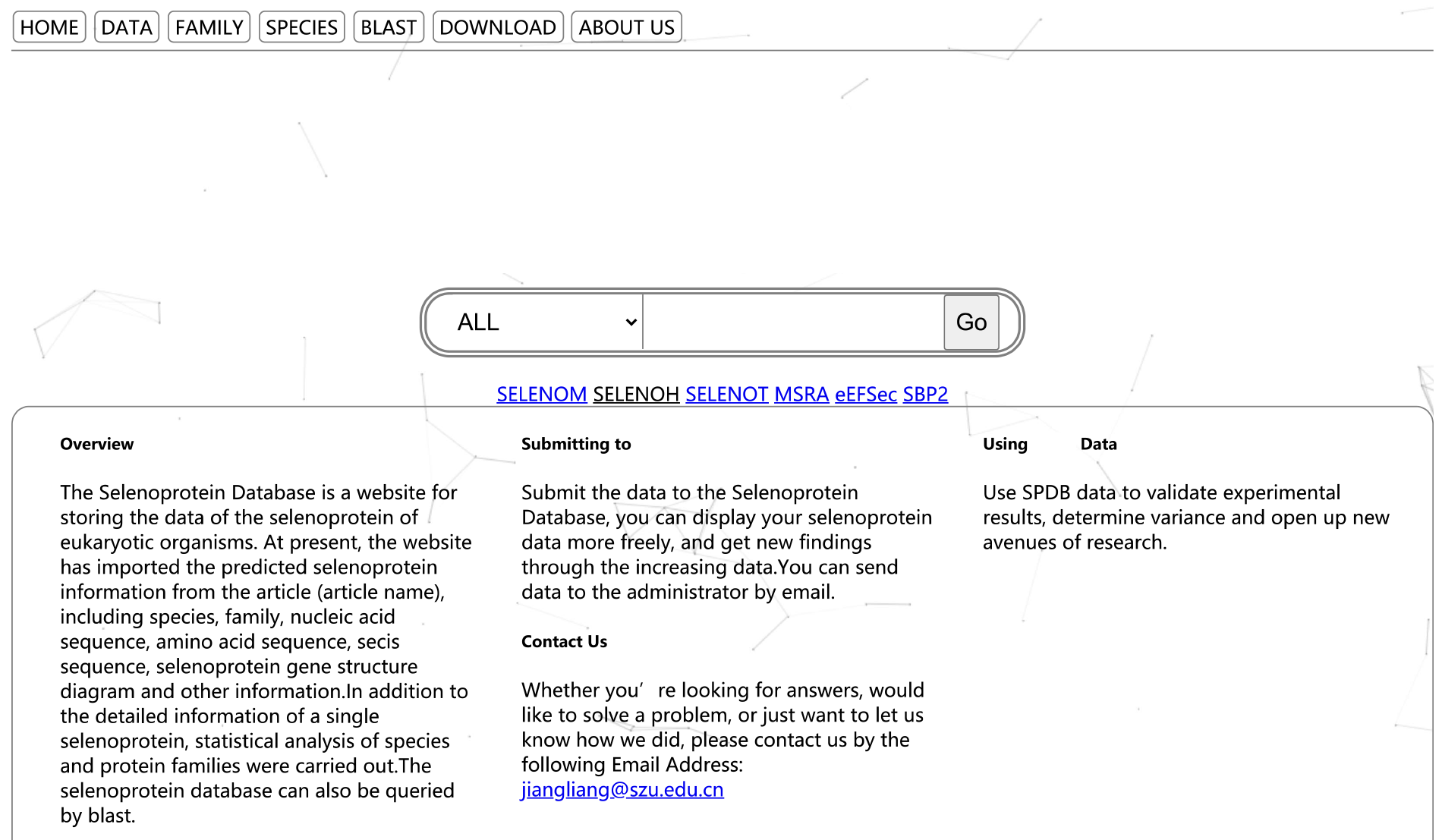


**Figure S****11 Keyword search page**

The keyword search is to use the user input species name, protein name and other information to search in the database. Currently, four keyword options of "ALL"," Species", "Protein Family" and "Protein ID" are provided. The "Species" option is to search the input information in the species, the "Protein Family" option is to search the input information in the protein family, the "Protein ID" option is to search the id recorded in the database, "ALL" is to search the data in the above three types of data, and "ALL" is the default option of the website. Records containing this data are searched simultaneously when the input data is searched. For example, filling in "Thalassiosira" in the search box will display the search results of "*Thalassiosira oceanica*" and "*Thalassiosira pseudonana*". The search results page will display partial information about the searched protein for users to preview, and click the corresponding ID to further browse the detailed information.


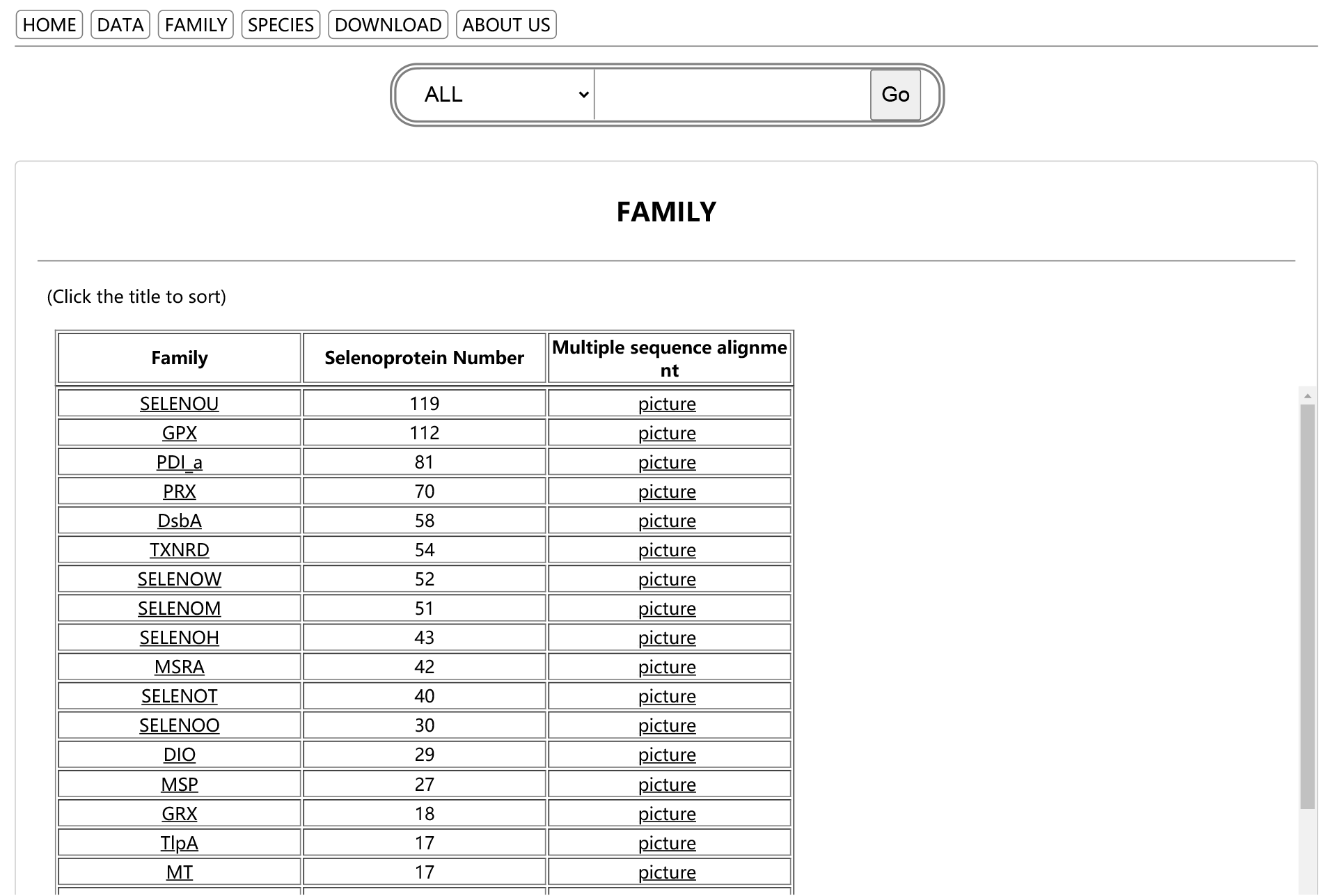


**Figure S12** **Selenoprotein family list search page**

List search is a more direct search, clearly shows the classification of data. At present, there are two lists of protein families and species. You can directly go to the corresponding result page by clicking the name of a protein family or species you want to search for. Click the family or species button at the top of the site to enter the corresponding list page.


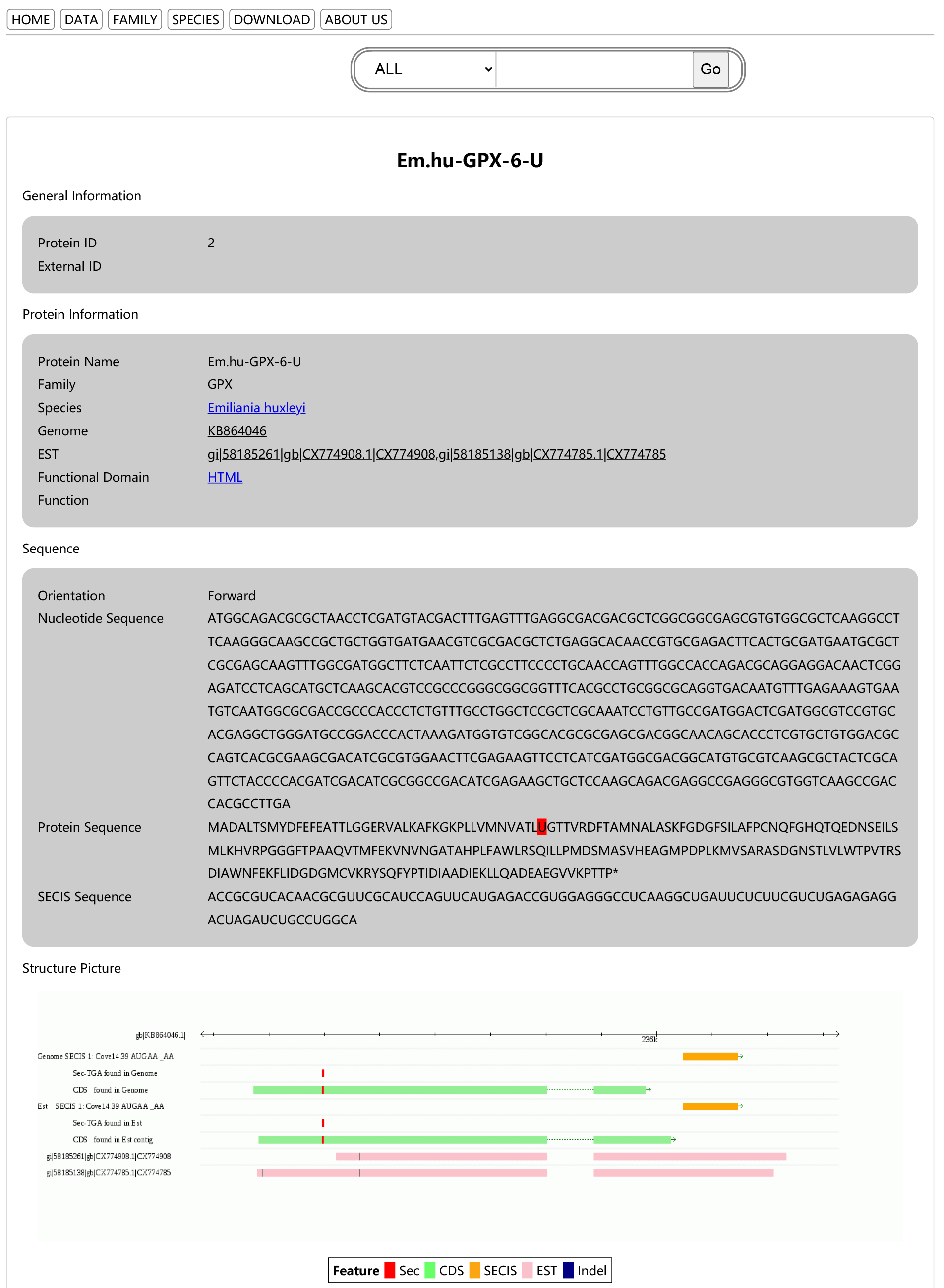


**Figure S1****3 Selenoprotein detailed information page**

In the SPDB website, annotation information of each selenoprotein includes protein name, genome information, EST information, protein sequence, gene sequence, SECIS element sequence, structural-functional domain, species, and functional information of the protein family. The page layout is shown in the picture. Among them, genome information, EST information and species information provide hyperlinks established with relevant pages of NCBI to facilitate users to consult and learn more detailed information. Protein Functional Domain Information can be entered into details through the hyperlink of Functional Domain in the Protein Information module. Moreover, the position of selenocysteine in the protein sequence is especially highlighted, so users can accurately find the selenocysteine in the protein sequence. Also, the Structure Picture provided a genome Structure map, which showed the sequence of selenoprotein, selenocysteine, and SECIS on the genome and EST data, and specifically identified the part of “indel” found in the sequence.


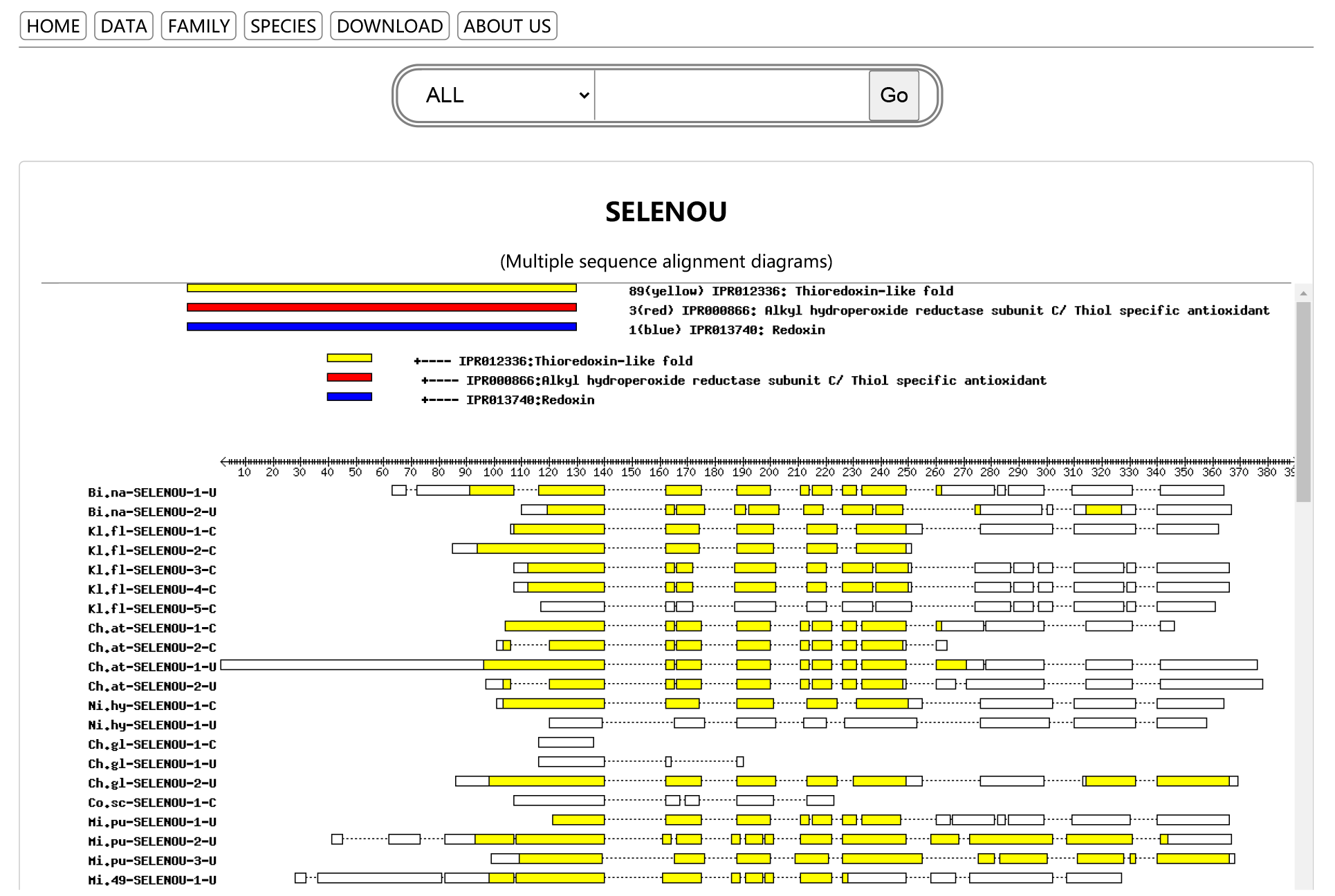


**Figure S14** **Multiple sequence alignments**

SPDB performed multiple sequence alignment for each selenoprotein family and visualized the alignment results. The user can find the multiple sequence alignment results of the family in the search list of the family page. Besides, the website statistics the distribution DATA of selenium protein of algae and generates a statistical chart, which can be downloaded from the DATA page. The selenoprotein dataset and database framework data are available for free download on the DOWNLOAD page.


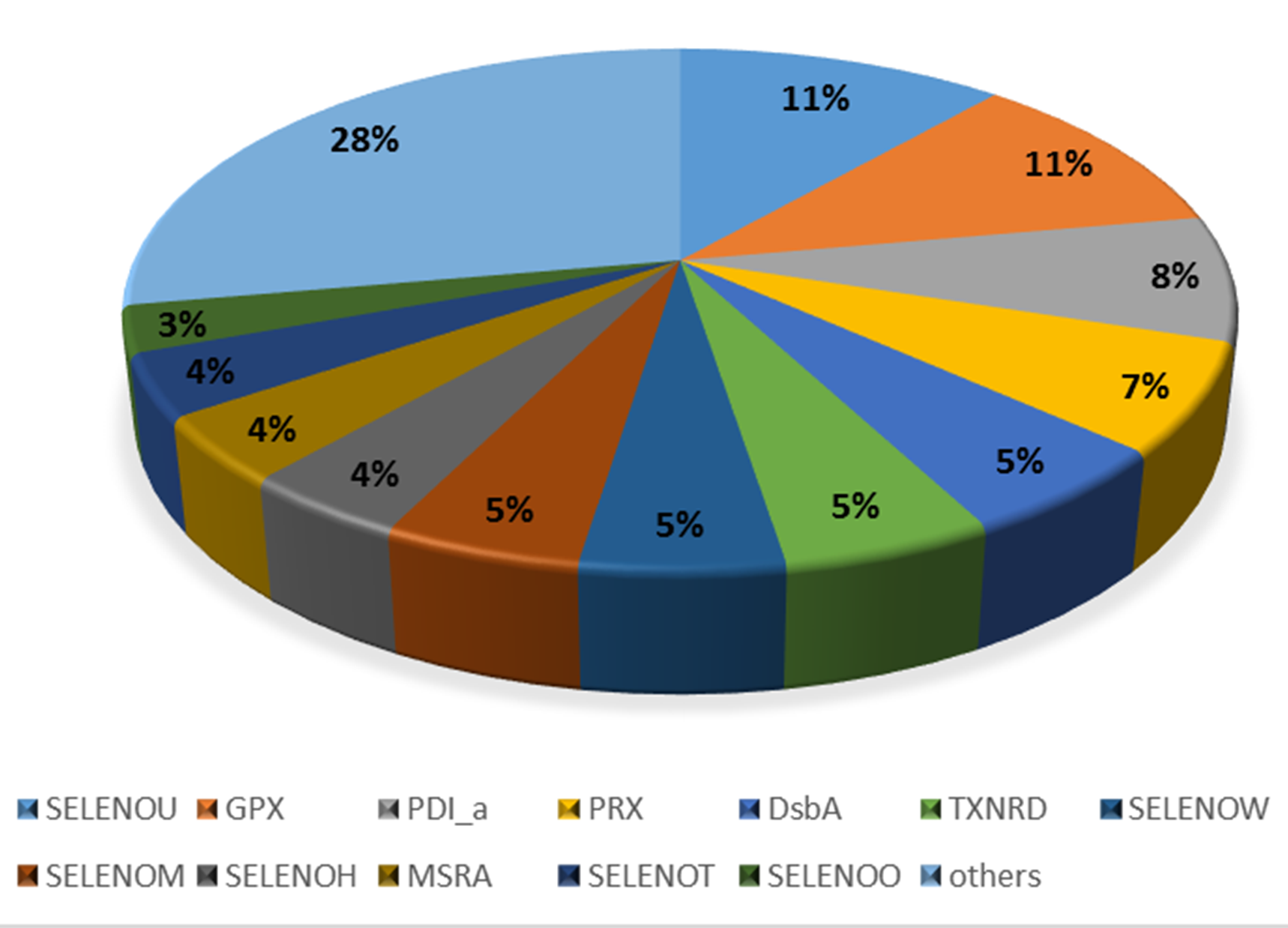


**Figure S15** **Selenoprotein family statistics in SPDB**

SPDB was established to provide better annotation information of protein, gene and SECIS element about selenoprotein. The database currently contains annotations for 36 species. The data on this website are all from the study and analysis of this article. The SPDB database contains 1041 data records, a total of 1041 selenoproteins. These selenoproteins covered 42 different selenoprotein families. As shown in the figure, SELENOU and GPX in SPDB are the two selenoprotein families with the highest proportion of the total database, and the two families account for 22%.
